# Supplementary material for: Maltreatment or violence-related injury in children and adolescents admitted to the NHS: comparison of trends in England and Scotland between 2005 and 2011
Source: BMJ Open. 2014 Apr 22;4(4):e004474. doi: 10.1136/bmjopen-2013-004474 (PMC4010840; doi:10.1136/bmjopen-2013-004474)

Maltreatment or violence-related injury in children and adolescents admitted to the NHS: comparison of trends in England and Scotland between 2005 and 2011

Journal:	*BMJ Open*
Manuscript ID:	bmjopen-2013-004474
Article Type:	Research
Date Submitted by the Author:	13-Nov-2013
Complete List of Authors:	Gonzalez-Izquierdo, Arturo; UCL Institute of Child Health, Centre of Paediatric Epidemiology and Biostatistics<br>Cortina Borja, Mario; UCL Institute of Child Health, General and Adolescent Paediatric Unit<br>Woodman, Jenny; Institute of Child Health, MRC Centre of Epidemiology for Child Health<br>Mok, Jacqueline; NHS Lothian University Hospitals Division (retired),<br>McGhee, Janice; University of Edinburgh, School of Social and Political Science<br>Taylor, Julie; University of Edinburgh, Child Protection Research Centre<br>Parkin, Chloe; UCL Institute of Child Health, Centre of Paediatric Epidemiology and Biostatistics<br>Gilbert, Ruth; UCL Institute of Child Health, Centre of Paediatric Epidemiology and Biostatistics
**Primary Subject Heading**:	Public health
Secondary Subject Heading:	Health policy, Paediatrics
Keywords:	Child protection < PAEDIATRICS, Non-accidental injury < PAEDIATRICS, Health policy < HEALTH SERVICES ADMINISTRATION & MANAGEMENT

SCHOLARONE™
Manuscripts

Title: Maltreatment or violence-related injury in children and adolescents admitted to the NHS: comparison of trends in England and Scotland between 2005 and 2011

Author list:

Arturo Gonzalez-Izquierdo[1], Mario Cortina-Borja[1], Jenny Woodman[1], Jacquie Mok[2], Janice McGhee[3], Julie Taylor[4], Chloe Parkin[1], Ruth Gilbert[1]

Author affiliations:

1	Dr Arturo Gonzalez-Izquierdo, Research Associate Statistician, arturo.gonzalez-izquierdo@ucl.ac.uk<br>Dr Mario Cortina-Borja, Senior Lecturer in Statistics, m.cortina@ucl.ac.uk<br>Jenny Woodman, PhD Research Student, j.woodman@ucl.ac.uk<br>Dr Chloe Parkin, Senior Research Associate, c.parkin@ucl.ac.uk<br>Professor Ruth Gilbert, Professor of Clinical Epidemiology, r.gilbert@ucl.ac.uk<br>Centre of Paediatric Epidemiology and Biostatistics, UCL Institute of Child Health, 30 Guilford Street, London WC1N 1EH, UK
2	Dr Jacqueline Mok, Lead Paediatrician in Child Protection, jacqui.mok@doctors.org.uk<br>Retired from clinical practice, formerly NHS Lothian University Hospitals Division
3	Janice McGhee, Senior Lecturer, Janice.McGhee@ed.ac.uk<br>School of Social and Political Science, University of Edinburgh
4	Julie Taylor, Professor of Child Protection, julie.taylor@ed.ac.uk<br>Child Protection Research Centre, University of Edinburgh, St Leonard's Land, Holyrood Road, Edinburgh EH8 8AQ

Corresponding author: Professor Ruth Gilbert, MRC Centre of Epidemiology for Child Health, UCL Institute of Child Health, 30 Guilford Street, London WC1N 1EH

Telephone: 0207 905 2101, FX: 0207 905 2793, e-mail: r.gilbert@ucl.ac.uk

3,404 words

For peer review only - http://bmjopen.bmj.com/site/about/guidelines.xhtml

KEY MESSAGES

What is already known:

- Trends in violence-related injury among adolescents have been decreasing in England and Scotland
- Scotland had higher rates of violence-related injury among adolescents than England in 2005

What this study adds:

- In Scotland between 2005 and 2011, maltreatment or violence-related injury admissions increased in infants but declined steeply among children aged 1-10 and 11-18 years along with declines in other injury admissions in all age groups.
- In England, rates of maltreatment or violence-related injury admission increased in infants and 1-10 year olds along with increases in other injury admissions, and declined in adolescents, though less steeply than in Scotland.

For peer review only

ABSTRACT (333 words)

Background Legislation to safeguard children from maltreatment by carers or violence by others was advanced in England and Scotland around 2004/5. Given differences in legislation, policies and services, we examined whether trends in injury admissions to hospital related to maltreatment or violence varied between the two countries.

Methods For children aged less than 19 years, we analysed rates of unplanned injury admission to National Health Service (NHS) hospitals in England and Scotland between Jan2005-Mar2012. We described differences in incidence trends for maltreatment or violence-related injury (MVR), comparing absolute rate differences between 2005 and 2011, and using Poisson and negative binomial regression models to estimate MVR-injury trends. We adjusted for seasonal effects and secular trends in non-MVR injury. Infants, children 1-10 years and adolescents 11-18 years were analysed separately.

Results In 2005, MVR-rates were similar in England and Scotland for infants and 1-10 year olds, but almost twice as high in Scotland for 11-18 year olds. MVR-rates for infants increased by similar amounts in both countries, in line with rising non-MVR rates in England but contrary to declines in Scotland. Among 1-10 year olds, MVR-rates increased in England and declined in Scotland, in line with increasing non-MVR rates in England and declining rates in Scotland. Among 11-18 year olds, MVR-rates declined in both countries along with declines in non-MVR trends but the decline was steeper in Scotland than in England.

Conclusion Differences in national child safeguarding policies implemented in 2004/05 may partly account for diverging MVR-trends between England and Scotland. Diverging trends may also reflect different investment in primary care, systems for reimbursement of hospital admissions or improved coordination of working relations between professionals in health and social care in Scotland. Similar trends in both countries for infants may reflect increased awareness of maltreatment, and for adolescents, policies to reduce violence.

For peer review only - http://bmjopen.bmj.com/site/about/guidelines.xhtml

STRENGTHS AND LIMITATIONS OF THIS STUDY

- Maltreatment or violence related injuries were analysed only if they resulted in admission to hospital. We could not determine to what extent children and adolescents with these injuries were being managed in other settings such as outpatient or emergency departments or primary care.
- Differences in coding practices, admission thresholds, performance targets, or allocation of resources could potentially influence trends.
- Strengths are that both countries have national health services and standardised, comparable hospital administrative data captured at a national level.
- A further strength is that analyses took into account background trends in admissions for other types of injuries, thereby partially accounting for admission thresholds related to system factors such as waiting times in the emergency department.

For peer review only

BACKGROUND

Exposure to maltreatment or violence is common during childhood. The term child maltreatment refers to any form of physical, emotional or sexual abuse or neglect that results in actual or potential harm to a child.[1,2] Among young children, maltreatment is mainly caused by carers. During adolescence, maltreatment more often results from violence by people other than carers, and includes assault or bullying by peers, siblings, other family members or strangers. Community surveys estimate that 40% to 60% of adolescents have been exposed to maltreatment by carers or abuse or violence by others during the previous year and around 1 in 10 of them would have been injured as a result.[3,4] Multiple exposures to abuse or violence, and from different sources, often co-occur in the same child. Moreover, multiple exposures to victimisation are strongly linked to adverse psychological outcome.[5,6]

A comparison between six developed countries showed an increase in contacts with child protection agencies but no decrease in the trends of other child maltreatment markers despite multiple policy initiatives targeting child protection.[7] It is known that children who live in deprived households, in a violent neighbourhood or whose parents are involved in violence or present serious mental illness or drug or alcohol misuse, are at increased risk of maltreatment or violence, whether perpetrated by their parents, other family members or people outside the family.[8-10] These risk factors suggest that policies affecting socioeconomic inequalities, social cohesion and antisocial behaviour and policies to safeguard children through improving services to disadvantaged families, might affect rates of maltreatment or violence.[11] In addition, policies that reduce risk factors for serious injury linked to maltreatment or violence, such as use of knives or other weapons, excessive alcohol consumption, and unregulated drug use, might reduce the risk of serious injury and thereby reduce admissions to hospital for maltreatment or violence-related (MVR) injuries.[12,13]

We conducted an ecological comparison of trends in hospital admissions for MVR injury admissions in England and Scotland from 2005, when both countries implemented major new legislation to safeguard children. We discuss the findings in relation to differences between the two countries in policies to safeguard children and the wider healthcare context.

For peer review only

For peer review only - http://bmjopen.bmj.com/site/about/guidelines.xhtml

METHODS

We analysed trends in monthly population incidence rates of unplanned MVR injury admission to hospital between 1st of January 2005 and 31st of March 2012 in England and Scotland. We used hospital administrative data for all NHS admissions of children in England (Hospital Episode Statistics – HES) and Scotland (Scottish Morbidity Records – SMR) to identify unplanned injury admissions using previously published methods (see Web-table 1 for definitions).[14,15] We defined MVR injury using a cluster of codes from the International Classification of Diseases (ICD10) recorded in any diagnostic field at discharge (up to 20 diagnostic fields per episode in HES or six diagnostic fields in SMR). Maltreatment or violence-related injury (MVR) codes were developed to be consistent with alert features mentioned in the National Institute of Health and Clinical Excellence (NICE) guidance for considering maltreatment and have been shown to produce similar trends with age and over time in previous cross-country comparisons.[7,16] The cluster of codes includes four subgroups (Web-table 1). These comprise specific references to maltreatment-syndrome, assault, unexplained injury, based on codes indicating the need for further evidence to determine the intent of injury (undetermined cause), and codes reflecting concerns about the child's social circumstances, family environment and adequacy of care; factors that in combination with an injury should alert clinicians to consider the possibility of maltreatment. We used admission rather than child, as the unit of analysis as very few children (<3%) had repeat maltreatment or violence-related injury admissions within a given year.

Denominator populations were derived from mid-year population estimates by year of age and calendar year published by the Office for National Statistics in England and the General Register Office for Scotland.[17,18] Analyses were stratified into three age groups reflecting broad stages of dependency, socialisation and exposure to violence (infants <1 year, children 1 to 10 years, and adolescents 11 to 18 completed years), which might be amenable to different policies. Maltreatment or violence-related injury in infancy is likely to reflect abuse or neglect by carers. Between 1 and 10 years of age, maltreatment or violence-related injury can reflect abuse or neglect by carers or inadequate protection of children from abuse or neglect by others. Among 11 to 18 year olds, physical violence by carers occurs at least as frequently as at younger ages, but violence due to other family members, peers, or strangers becomes much more frequent than that perpetrated by carers.[6,19]

Analyses

We plotted monthly incidence rates using three-monthly moving average rates. We used time series analyses and fitted segmented-Poisson and negative binomial regression models (generalised linear models) to determine trends and the possible timing of changes in gradient. [20-23] We took account of underlying trends in injury admission rates by adjusting analyses for unplanned injury

For peer review only - http://bmjopen.bmj.com/site/about/guidelines.xhtml

admissions that were not related to maltreatment or violence (non-MVR). We also fitted sine and/or cosine terms to account for annual seasonal variation; changes in the goodness-of-fit produced by including these periodic components were measured by the Akaike's information criterion (AIC). To determine whether trends significantly changed direction over time, we fitted segmented models with up to one change point. Negative binomial regression models were considered to account for over-dispersion as the variance of MVR injury rates is likely to increase for increasing rate values. We compared goodness-of-fit across nested Poisson and negative binomial models using the log-likelihood ratio test.

Because of the relatively large number of parameters in the models and limited power resulting from small numbers of monthly incidence points, we analysed each country separately and report qualitative differences. In each country we estimated absolute differences between rates in calendar years 2005 and 2011 within each age group for MVR and non-MVR injury incidence rates. We also report gradients given by the change point models (and their 95% confidence intervals), and the *p*-values associated with the gradients and, where relevant, with the change in gradient during the study period. A *p*-value <0.05 was considered significant. We also plotted smoothed incidence rates predicted by the model, using a non-parametric adaptive-smoother.[24] Regression models and nonparametric smoothers were fitted using the R environment for statistical computing, version 2.14.2 (http://www.R-project.org).[25] We used the R packages MASS[26] and SiZer[27] to fit piecewise linear models for Poisson and negative binomial distributions.

For peer review only

For peer review only - http://bmjopen.bmj.com/site/about/guidelines.xhtml

RESULTS

There were 54,207 MVR injury admissions in England and 7,367 in Scotland during the study period (Jan 2005 – Mar 2012). MVR injury admission rates were distributed similarly across age groups in both countries, with the vast majority occurring in adolescents 11-18 years, (78.2% in England and 88.3%in Scotland; Table 1). The age-related incidence of MVR injury admission was J-shaped with a subsidiary peak in infancy and a major peak in late adolescence (Table 1). Figure 1 A,B,C shows MVR incidence trends in England and Scotland by age group. Negative binomial models performed significantly better in all age groups (*p*-value < 0.05); except for the analysis of rates in Scottish infants where the corresponding Poisson model is reported (Figure 1 and Web-table 2). With the exception of infants, monthly trends showed marked seasonal patterns in both countries with peaks in the spring-summer months.

For infants, the incidence of MVR injury admission increased between 2005 and 2011 in both countries: in England 27.8 and in Scotland 23.5 per 100,000 more infants were admitted for MVR injury in 2011 compared with in 2005 (Table 2, Figure 1A). This represented an increase of 37% from 2005 rates in England and of 23% in Scotland (Figure 2). The increase in England appears to have been largely determined by the background increase in non-MVR injury admissions as adjustment for the non-MVR trend and seasonal variation showed a 6.6% annual increase that was not significant at the 5% level.

In Scotland, the 18% annual increase in MVR admissions among infants estimated by the multivariable model did not reach a significant level (*p*=0.065) and was in contrast to the decline in non-MVR injury admissions (Figure 1, Web-table 2). The absolute increase between 2005 and 2011 in admission rates for non-MVR injury in England and decrease in Scotland is shown in Table 2 and Figure 2. Trends for non-MVR injury are shown in Web-figure 1A.

Among children aged 1-10 years, the incidence of MVR injury admission increased between 2005 and 2011 in England (rate difference 7.8/100,000) and declined similarly in Scotland (rate difference 9.9/100,000; Table 2, Figure 1B). This represented an increase of 50% from 2005 rates in England, and a decrease of 50% in Scotland (Figure 2). Using multivariable models to adjust for trends in non-MVR injury admissions and seasonal variation, we estimated diverging annual incidence trends: rates were increasing by 10.4% per year in England and declining by 10.8% per year in Scotland. Both these trends were significant at the 5% level (Figure 1B, Web-table 2). In this age group, admissions for non-MVR injury increased in England and decreased in Scotland (Table 2 and Web-figure 1B).

For peer review only - http://bmjopen.bmj.com/site/about/guidelines.xhtml

Among adolescents aged 11-18 years, admission rates for MVR injury in 2005 were almost twice as high in Scotland as in England (Table 2, Figure 1C). A steep decline from the autumn of 2006 in Scotland resulted in converging rates in the two countries by 2011 as rates in England declined more slowly (Figure 1C). The absolute difference in rates between 2005 and 2011 resulted in 15.8/100,000 fewer adolescents admitted with MVR injury in 2011 in England and 85.2/100,000 fewer in Scotland, relative reductions of 13.3% and 41.4% respectively (Table 2, Figure 2, Web-table 2). These trends were steeper than the declining trends in non-MVR admissions in both countries and were significant at the 5% level after adjusting for trends in non-MVR injury admissions and seasonal variation (Web-table 2). We estimated an annual decline in the incidence of MVR injury admissions in England of 7.5%, which dated from 2010. The decline for 11-18 year olds in Scotland was steeper (12.9%) and dated from 2006 (Figure 1C, Web-table 2). The rate of admission for non-MVR injury declined similarly in both countries (Figure 2, Web-figure 1C).

For peer review only

For peer review only - http://bmjopen.bmj.com/site/about/guidelines.xhtml

DISCUSSION

Between 2005 and 2011 rates of maltreatment or violence-related injury admission increased in England among infants and 1-10 year olds along with rises in other injury admissions and declined in adolescents, though less steeply than in Scotland. Maltreatment or violence-related injury admissions in Scotland increased in infants but declined steeply among children aged 1-10 and 11-18 years along with declines in other injury admissions in all age groups. Similarities between England and Scotland were increasing rates of MVR injury admissions among infants and decreasing rates among 11-18 year olds.

Among 1-10 year olds, incidence trends for MVR injury admissions diverged between England (increasing) and Scotland (decreasing), but were consistent with trends in other injuries in this age group. Among 11-18 year olds, rates of MVR injury admission were twice as high in Scotland as in England in 2005, but fell more steeply than in England, resulting in similar rates by 2011.

A limitation of the study is that changes in diagnostic coding practices could potentially account for differing trends over time in England and Scotland. Maltreatment or violence-related injury admissions have been found to be highly specific but relatively insensitive.[28-30] Improvements in sensitivity of coding might account for increases in England where coding depth is incentivised by the remuneration system 'payment by results', which does not operate in Scotland.[31,32] An alternative explanation for the declining rates of MVR injury admissions among 11-18 year olds could be a diagnostic drift, whereby codes for related presentations, such as drug or alcohol misuse which may co-occur with presentations such as assault in adolescents, are coded instead of assault. This explanation is unlikely, as coding depth tends to increase over time.

Changes in admission thresholds could differentially affect rates in both countries. We confined our analyses to admissions, rather than emergency departments (ED) or primary care because of the availability of coded data. Attendances at the ED for injury outnumber admissions for injury by around 10 to 1.[33] Flows of patients from the ED to short stay admissions may have increased following introduction of four-hour wait targets in the ED.[34] However, these targets were implemented in both Scotland and England in 2004, though in England the target was dropped in 2010 from 98% waiting less than 4 hours to 95%.[35] Alternative explanations for the decline in incidence of non-MVR injury admissions in all age groups in Scotland, in contrast to increases in England for children aged less than 10 years, include differences in injury occurrence or different thresholds for admission, possibly linked to better coordination of primary care support in Scotland.

Management of children with suspected maltreatment or neglect is also changing, particularly in Scotland, where many children with suspected maltreatment are neither admitted to hospital nor attend emergency departments. Closer working between health and social care professionals have

For peer review only - http://bmjopen.bmj.com/site/about/guidelines.xhtml

resulted in a large proportion of children receiving medical assessments in out-patient clinics.[36] Many are also being seen by general practitioners.[37-39] Within the NHS, community paediatricians have taken on a major role as ‘child abuse paediatricians’ and are referred children for assessments in clinics held either within or outside the hospital setting.[36] Hence small changes in admission thresholds for injury, or for specific subgroups such as those with maltreatment or violence, could have a substantial impact on rates of admission.

The broader service context may also be relevant. Scotland spends more on the NHS than England and has more GPs per capita.[40] Approaches to configuration of services post devolution have been characterised as focussing on markets and management in England and on the medical profession and cooperation in Scotland.[41] In addition, Scotland abolished the purchaser/provider split and the idea of provider competition, and recreated organisations responsible for meeting the needs of the population and running services within defined geographical areas. This may have made it easier to integrate and coordinate services, and therefore improve quality of care along the patient pathway.[40]

Policies related to child maltreatment or violence

To identify policies that may have influenced trends in MVR injury admissions, we asked researchers in England and Scotland to independently list policy initiatives related to child maltreatment or violence that applied nationally in each country, without knowledge of the results of the trend analyses. Relevant legislation, government strategies, inter-agency guidance, implementation or accountability frameworks or health guidance documents were included, but research evidence and guidance by professional bodies were excluded. The results, summarised in Web-table 3, show at least one set of guidance published in one or both countries every year since 2002.

Both countries saw comparable ‘integrative’ policies initiated during the first half of the 2000s. “Every child matters” in England and “Getting it right for every child” – GIRFEC – in Scotland, aimed to promote cooperation across sectors and agencies working for the wellbeing of children.[42] However, implementation differed between the two countries. In England, ‘Every Child Matters’ introduced a new structure of children’s centres to coordinate targeted and specialist services, separately from existing services.[43] In contrast, GIRFEC promoted coordination within existing structures to integrate universal and targeted services.[44] It is hard to relate the timing of implementation of these two major policy initiatives to changes in trends observed in our study as implementation took place over unspecified times. For example, GIRFEC was launched in 2005 but took several years to be fully implemented.[45,46]

For peer review only - http://bmjopen.bmj.com/site/about/guidelines.xhtml

The increase in the incidence of MVR injury admissions among infants in England and Scotland may reflect raised awareness of maltreatment in infants. However, alternative explanations, such as coding practices or a true increase cannot be ruled out. The increase in England predated 2008, when two events occurred that are likely to have raised awareness of maltreatment related injuries presenting to healthcare. The first was NICE guidance '*When to suspect child maltreatment*',[47] which was mandated for hospitals in England but not in Scotland. This guidance coincided with extreme media publicity between October 2008 and August 2009 of the death of Peter Connelly in north London, a 17 month old child who died from more than 50 injuries inflicted by his parents. These events may have influenced the increase in MVR injury admissions among infants in Scotland, which dated from April 2009, and was in contrast to declines in non-MVR injury admissions among infants.

Among 1-10 year olds, diverging trends in the incidence of MVR injury admissions, with increasing rates from 2007 in England and decreasing rates from 2005 in Scotland, persisted after adjusted for background trends in non-MVR injury admissions. Presentation with MVR injuries in this age group may be truly declining in Scotland and increasing in England. On the other hand, admission rates are highly susceptible to policies about whether, when and where to investigate possible child maltreatment. For example, we have previously reported steep declines in maltreatment-related injury admissions in Manitoba, Canada, following a change in policy to investigate possible maltreatment in the community, avoiding admission to hospital.[7] The fact that Scotland has seen a concurrent decline in referrals to the Scottish Children's Reporter Administration over the same period[48] could indicate a true decline in child maltreatment or active management in the community without referral of cases to hospital.

The decline in MVR injury admissions among 11-18 year olds in England and Scotland, despite adjusting for background trends in non-MVR injuries, could be related to admission thresholds for assault. Alternatively, these decreasing trends could reflect true declines in MVR injury serious enough to require admission. A true decline would be consistent with falls in violent crime reported in police statistics. The steeper and more prolonged decline in MVR injury admissions in Scotland compared with England may reflect intensive programmes to prevent youth violence and reduce drug and alcohol misuse.[49,50] Both England and Scotland implemented the 'challenge 25' policy in 2009 to reduce youth access to alcohol.[51]

Implications

Our analyses show that the incidence of MVR injury admissions in children can change substantially over time and in opposite directions in adjacent countries with similar healthcare systems. The declines in Scotland suggest that the increasing rates observed in England are not inevitable. However, which policies, if any, have influenced these changes cannot be determined from this

For peer review only - http://bmjopen.bmj.com/site/about/guidelines.xhtml

study. A priority for future research is to distinguish true change in the occurrence of MVR injury needing admission from changes in admission thresholds. This requires analyses of all cases of MVR injury presenting to primary care, those seen as outpatients by community paediatricians, those attending the emergency department and those admitted to hospital, to understand how children are managed within the healthcare system. Such analyses are not yet possible due to the lack of well-coded, administrative healthcare databases across health sectors.

Hospitalisation for maltreatment-related injury or injury due to other forms of victimisation represents considerable suffering to the child and a major cost to the health service. These results strengthen the call by WHO to use administrative data to improve understanding of how policy can reduce exposure of children to injury due to violence or neglect.[52] The need for improved data collection of activity by community paediatricians is also highlighted.

For peer review only - http://bmjopen.bmj.com/site/about/guidelines.xhtml

Licence for publication

The corresponding author has the right to grant on behalf of all authors and does grant on behalf of all authors, an exclusive licence on a worldwide basis to the BMJ Publishing Group Ltd and its licensees, to permit this article (if accepted) to be published in BMJ editions and other BMJPG products and to exploit all subsidiary rights, as set out in our licence (http://resources.bmj.com/bmj/authors/checklists-forms/licence-for-publication)

Competing interests

All authors have completed the Unified Competing Interest form at www.icmje.org/coi_disclosure.pdf (available on request from the corresponding author). AGI, MCB, JW, JM, JMG, JT, CP and RG have no competing interests that may be relevant to the submitted work.

Authors' contributions

RG and AGI conceived the paper and the analytic plan. AGI wrote the first draft and carried out the analyses. RG wrote the final version. All authors commented on the analyses and report. RG is guarantor.

Acknowledgment

AGI was supported by funding from the Department of Health Policy Research Programme through funding to the Policy Research Unit in the Health of Children, Young People and Families. The views expressed are not necessarily those of the Department. We would like to thank members of the Policy Research Unit for the health of children, young people and families: Terence Stephenson, Catherine Law, Amanda Edwards, Ruth Gilbert, Steve Morris, Helen Roberts, Catherine Shaw, Russell Viner and Miranda Wolpert. We are grateful to Andrew Woolley for commenting on the policy timeline for England.

This work is supported by awards establishing the Farr Institute of Health Informatics Research at UCLP Partners from the MRC, in partnership with Arthritis Research UK, the British Heart Foundation, Cancer Research UK, the Economic and Social Research Council, the Engineering and Physical Sciences Research Council, the National Institute of Health Research, the National Institute for Social Care and Health Research (Welsh Assembly Government), the Chief Scientist Office (Scottish Government Health Directorates) and the Wellcome Trust (MR/K006584/1).

Transparency declaration

Ruth Gilbert on behalf of all co-authors affirms that this manuscript is an honest, accurate, and transparent account of the study being reported; that no important aspects of the study have been

For peer review only

omitted; and that any discrepancies from the study as planned (and, if relevant, registered) have been explained.

Data sharing

Additional data can be accessed in the web-appendix. Source data can be accessed by researchers applying to the Information Services Division Scotland or the Health and Social Care Information Centre for England.

For peer review only

For peer review only - http://bmjopen.bmj.com/site/about/guidelines.xhtml

Reference List

1. World Health Organization. Health Topics. Child maltreatment. 2013. WHO. 2013.

2. HM Government. Working together to safeguard children. A guide to interagency working to safeguard and promote the welfare of children. 2013. London, Department for Education.

3. Finkelhor D, Turner H, Ormrod R, Hamby SL: Violence, abuse, and crime exposure in a national sample of children and youth. Pediatrics 2009, 124: 1411-1423.

4. Radford L, Corral S, Bradley C, Fisher H, Bassett C, Howat N et al.. Child abuse and neglect in the UK today. 2011. London, NSPCC.

5. Finkelhor D, Ormrod RK, Turner HA: Polyvictimization and trauma in a national longitudinal cohort. Dev Psychopathol 2007, 19: 149-166.

6. Finkelhor D, Ormrod RK, Turner HA: The developmental epidemiology of childhood victimization. J Interpers Violence 2009, 24: 711-731.

7. Gilbert R, Fluke J, O'Donnell M, Gonzalez-Izquierdo A, Brownell M, Gulliver P et al.: Child maltreatment: variation in trends and policies in six developed countries. Lancet 2012, 379: 758-772.

8. Finkelhor D, Ormrod R, Turner H, Holt M: Pathways to poly-victimization. Child Maltreat 2009, 14: 316-329.

9. De VM, Demyttenaere K, Bruffaerts R: The relationship between adverse childhood experiences and mental health in adulthood. A systematic literature review. Tijdschr Psychiatr 2013, 55: 259-268.

10. Farrington DP, Loeber R: Epidemiology of juvenile violence. Child Adolesc Psychiatr Clin N Am 2000, 9: 733-748.

11. Murray J, Farrington DP: Risk factors for conduct disorder and delinquency: key findings from longitudinal studies. Can J Psychiatry 2010, 55: 633-642.

12. Norman RE, Byambaa M, De R, Butchart A, Scott J, Vos T: The long-term health consequences of child physical abuse, emotional abuse, and neglect: a systematic review and meta-analysis. PLoS Med 2012, 9: e1001349.

13. Statham J: Effective services to support children in special circumstances. Child Care Health Dev 2004, 30: 589-598.

14. NHS National Services Scotland - Information Services Division. Health and Social Care Data Dictionary - SMR01 General/ Acute Impatient and Day Cases. 2012.

15. NHS.The Information Centre for Health and Social Care. HES Online. What is the data? 2012.

For peer review only - http://bmjopen.bmj.com/site/about/guidelines.xhtml

16. Gonzalez-Izquierdo A, Ward A, Donnell O, Li L, Roposch A, Stanley F et al.: Cross-country comparison of victimisation-related injury admission in children and adolescents in England and Western Australia. BMC Health Serv Res 2013, 13: 260.

17. General Register Office for Scotland. Mid-Year Population Estimates. 2012.

18. Office for National Statistics. Population Estimates for UK, England and Wales, Scotland and Northern Ireland. 2012.

19. Radford L, Corral S, Bradley C, Fisher H, Bassett C, Howat N et al.. Child cruelty in the UK: An NSPCC study into child abuse and neglect over the past 30 years. 2011. London, NSPCC.

20. Biglan A, Ary D, Wagenaar AC: The Value of Interrupted Time-Series Experiments for Community Intervention Research. Prevention Science 2000, 1: 31-49.

21. Toms JD, Lesperance ML: Piecewise regression: a tool for identifying ecological thresholds. Ecology 2003, 84: 2034-2041.

22. Wagner AK, Soumerai SB, Zhang F, Ross-Degnan D: Segmented regression analysis of interrupted time series studies in medication use research. Journal of Clinical Pharmacy and Therapeutics 2002, 299-309.

23. Hilbe JM: Negative binomial regression, Second Edition edn. Cambridge: Cambridge University Press; 2011.

24. Friedman JH. A variable span scatterplot smoother. Edited by Laboratory for Computational Statistics. 5. 1984. Standford University. Technical Report No. 5.

25. R Development Core Team. R: A language and environment for statistical computing. 2008. Vienna, R Foundation for Statistical Computing.

26. Venables WN, Ripley BD: Modern Applied Statistics with S, Fourth Edition edn. Springer; 2002.

27. Sonderegger DL, Wang HN, Clements WH, Noon BR: Using SiZer to detect thresholds in ecological data. Frontiers in Ecology and Environment 2009, 7: 190-195.

28. Gonzalez-Izquierdo A, Woodman J, Copley L, van der Meulen J, Brandon M, Hodes D et al.: Variation in recording of child maltreatment in administrative records of hospital admissions for injury in England, 1997-2009. Arch Dis Child 2010, 95: 918-925.

29. McKenzie K, Scott D, Waller G, Campbell M: Reliability of Routinely Collected Hospital Data for Child Maltreatment Surveillance. BMC Public Health 2011, 11: 8.

30. Gonzalez-Izquierdo A, Ward A, Smith P, Walford C, Begent J, Ioannou Y et al.: Notifications for child safeguarding from an acute hospital in response to presentations to healthcare by parents. Child: care health and development, 2013, Under review.

31. Audit Commission. Payment by results assurance framework. 2006. Audit Commission.

For peer review only

For peer review only - http://bmjopen.bmj.com/site/about/guidelines.xhtml

32. Jameson S, Reed MR: Payment by results and coding practice in the National Health Service. The importance for orthopaedic surgeons. J Bone Joint Surg Br 2007, 89: 1427-1430.

33. Woodman J, Pitt M, Wentz R, Taylor B, Hodes D, Gilbert R: Performance of screening tests for child physical abuse in Accident and Emergency Departments. Health Technol Assess 2008, 12: iii, xi-xiii 1-95.

34. Gill PJ, Goldacre MJ, Mant D, Heneghan C, Thomson A, Seagroatt V et al.: Increase in emergency admissions to hospital for children aged under 15 in England, 1999-2010: national database analysis. Arch Dis Child 2013, 98: 328-334.

35. Appleby J: Are accident and emergency attendances increasing? BMJ 2013, 346: f3677.

36. Kirk CB, Lucas-Herald A, Mok J: Child protection medical assessments: why do we do them? Arch Dis Child 2010, 95: 336-340.

37. Woodman J, Allister J, Rafi I, de Lusignan S, Belsey J, Petersen I et al.: A simple approach to improve recording of concerns about child maltreatment in primary care records: developing a quality improvement intervention. Br J Gen Pract 2012, 62: e478-e486.

38. Woodman J, Freemantle N, Allister J, de LS, Gilbert R, Petersen I: Variation in recorded child maltreatment concerns in UK primary care records: a cohort study using The Health Improvement Network (THIN) database. PLoS One 2012, 7: e49808.

39. Woodman J, Gilbert RE, Allister J, Glaser D, Brandon M: Responses to concerns about child maltreatmentt: a qualitative study of GPs in England. BMJ Open 2013, in press.

40. Connolly S, Bevan G, Mays N. Funding and performance of healthcare systems in the four UK countries before and after devolution. 2010. London, The Nuffield Trust.

41. Greer S. Four Way Bet: How devolution has led to four different models for the NHS. 2004. London, The Constitution Unit.

42. Stafford A, Parton N, Vincent S, Smith C: Child protection systems in the United Kingdom. A comparative analysis. London, UK: Jessica Kingsley Publishers; 2012.

43. Department for Education. Every child matters. Chapter 5 Accountability and integration. 2003. The Stationary Office.

44. The Scottish Goverment. A guide to Getting it right for every child. Chapter 2 Getting it right in practice. 2012. The Scottish Goverment.

45. Children's Hearings System. Getting it right for every child. Proposals for action. 2005. Scottish Executive.

46. The Scottish Government. Getting it right for children and families. A guide to getting it right for every child. 2012.

47. National Collaborating Centre for Women's and Children's Health. When to suspect child maltreatment . 2009. London, National Institute for Health and Clinical Excellence.

For peer review only

For peer review only - http://bmjopen.bmj.com/site/about/guidelines.xhtml

48. McGhee J, McGhee J: Children's Hearings in Scotland: Balancing Rights and Welfare. In Social Work and the Law in Scotland. Social Work and the Law in Scotland. Edited by Gordon J, Gordon J. Milton Keynes: The Open University and Basingstoke: Palgrave Macmillan; 2011:85-100.

49. The Scottish Government. Youth Justice. 2013. The Scottish Goverment. 2013.

50. Gov.UK. Youth crime prevention programmes. 2013. Gov.UK. 2013.

51. Drink Aware. Buying alcohol. 2013. Drink Aware.

52. WHO. World Report on Violence and health. 1-372. 2002.

For peer review only

For peer review only - http://bmjopen.bmj.com/site/about/guidelines.xhtml

Table 1 Hospital admissions for maltreatment or violence-related (MVR) injury in children in England and Scotland between 2005 and 2011, inclusive

Country	Age group	Total unplanned injury	Unplanned injury rates per 100,000 cy *	MVR injury	MVR incidence rates per 100,000cy *	% of total MVR injury**	% of total injury ***
England	<1y	61,987	1361.3	3,955	86.9	7.3	6.4
	1-10y	402,334	962.5	7,876	18.8	14.5	2.0
	11-18y	414,259	1157.4	42,376	118.4	78.2	10.2
	Subtotal	878,580	1069.5	54,207	66.0	100	6.2
Scotland	<1y	5,168	1277.9	290	71.7	3.9	5.6
	1-10y	48,357	1244.3	570	14.7	7.7	1.2
	11-18y	51,339	1471.0	6,507	186.4	88.4	12.7
	Subtotal	104,864	1347.7	7,367	94.7	100	7.0

* Denominators are the mid-year population estimates from Office for National Statistics and General Register Office for Scotland, cy = child years
** Denominator is the total number of maltreatment or violence-related injury admissions in children 0 to 18 years of age by country
*** Denominator is the total number of unplanned injury admissions within age group & country

For peer review only

For peer review only - http://bmjopen.bmj.com/site/about/guidelines.xhtml

Table 2 Observed mean incidence rate per 100,000 children in calendar years 2005 and 2011 and absolute difference in rates

		Rate (95%CI) **2005**		Rate (95%CI) **2011**		Absolute difference in rates (95%CI)	
Country	Age group	MVR	Non-MVR	MVR	Non-MVR	MVR	Non-MVR
England	<1y	75.0 (68.1, 81.9)	1146.9 (1120.0, 1173.9)	102.8 (95.2, 110.4)	1334.7 (1307.2, 1362.2)	27.8 (27.1, 28.5)	187.8 (187.2, 188.3)
	1-10y	15.5 (14.5, 16.5)	909.6 (901.9, 917.3)	23.3 (22.1, 24.5)	950.6 (942.9, 958.2)	7.8 (7.6, 8.0)	41.0 (41.0, 40.9)
	11-18y	119.1 (116.2, 122.1)	1076.5 (1067.6, 1085.4)	103.3 (100.6, 106.1)	896.2 (888, 904.5)	-15.8 (-15.6, -16.0)	-180.3 (-179.6, -180.9)
Scotland	<1y	66.1 (44.5, 87.7)	1426.3 (1326.0, 1526.6)	89.6 (65.5, 113.7)	1024.1 (942.5, 1105.6)	23.5 (21.0, 26.0)	-402.3 (-383.5, -421.0)
	1-10y	19.6 (16.0, 23.3)	1306.2 (1276.2, 1336.3)	9.7 (7.2, 12.3)	1136.6 (1108.8, 1164.3)	-9.9 (-8.8, -11.0)	-169.7 (-167.4, -172.0)
	11-18y	205.8 (193.4, 218.2)	1314.3 (1283.0, 1345.7)	120.6 (110.8, 130.5)	1113.7 (1083.8, 1143.7)	-85.2 (-82.6, -87.7)	-200.6 (-199.2, -202.0)

For peer review only

For peer review only - http://bmjopen.bmj.com/site/about/guidelines.xhtml

Figure 1 Monthly incidence trends from Jan 2005 to Mar 2012 of MVR injury in A) Infants, B) Children aged 1-10 years, and C) Adolescents aged 11-18 years, in England (dark grey**) and Scotland (**light grey**)**. Faint lines represent observed rates and bold lines represent three-monthly moving averages. Dashed lines represent smoothed trends of incidence rates estimated from the segmented regression analysis (except for trends in Scottish 1-10 year-olds where a standard Poisson regression was used) and markers indicate the change point estimated by the segmented regression model.

Figure 2 Absolute rate difference between annual incidence rates in calendar years 2005 and 2011 for MVR injury and non-MVR injury admissions of children and adolescents by age group and country.

Footnote for the figure: % change measured as (absolute difference)/(rate in 2005)

For peer review only

For peer review only - http://bmjopen.bmj.com/site/about/guidelines.xhtml

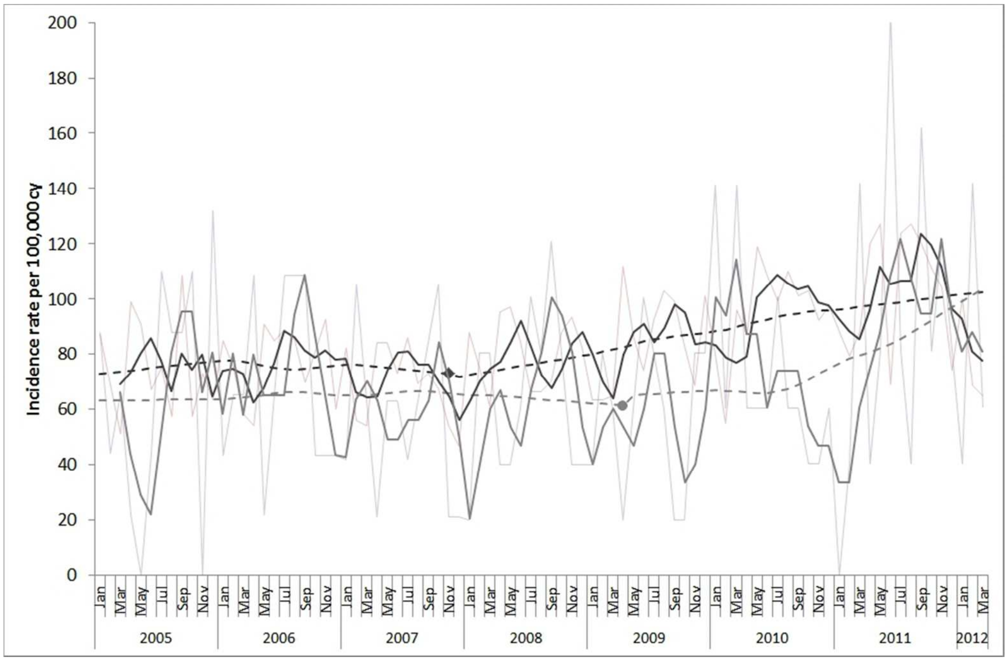

258x168mm (96 x 96 DPI)

For peer review only

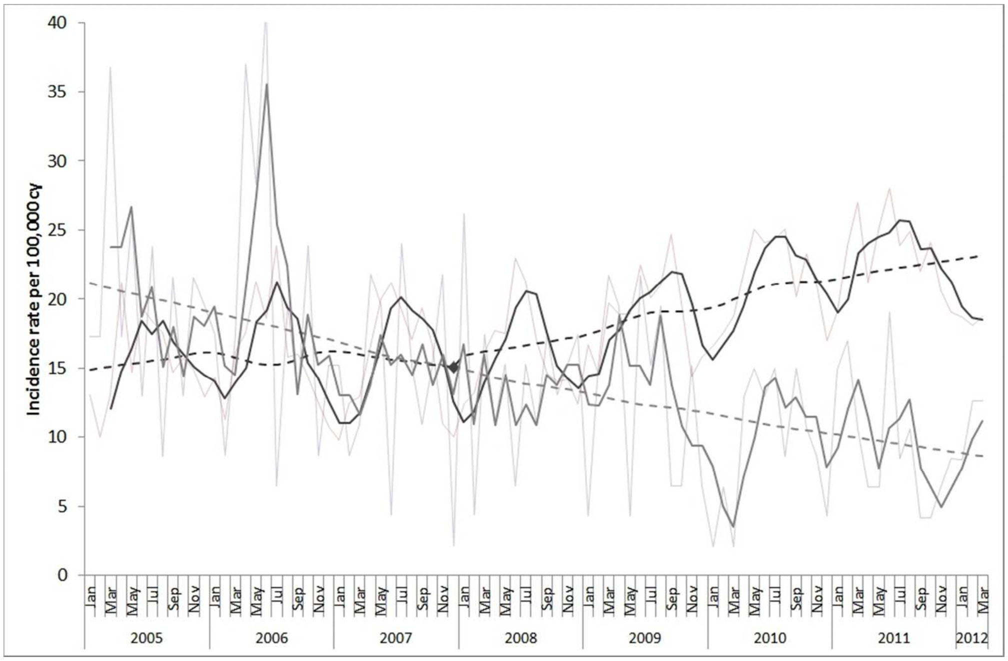

258x168mm (96 x 96 DPI)

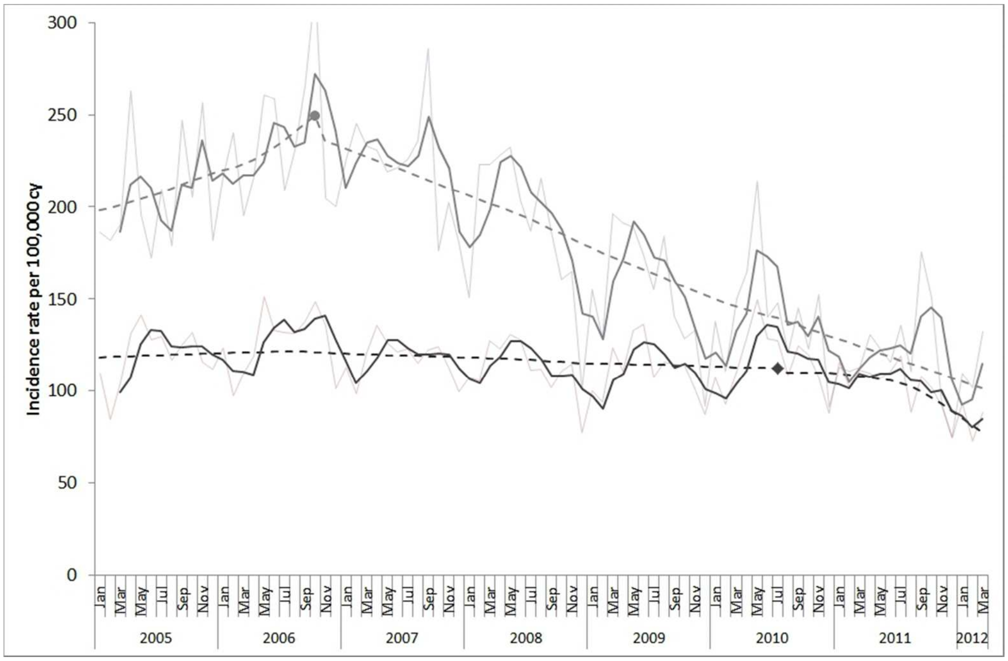

258x168mm (96 x 96 DPI)

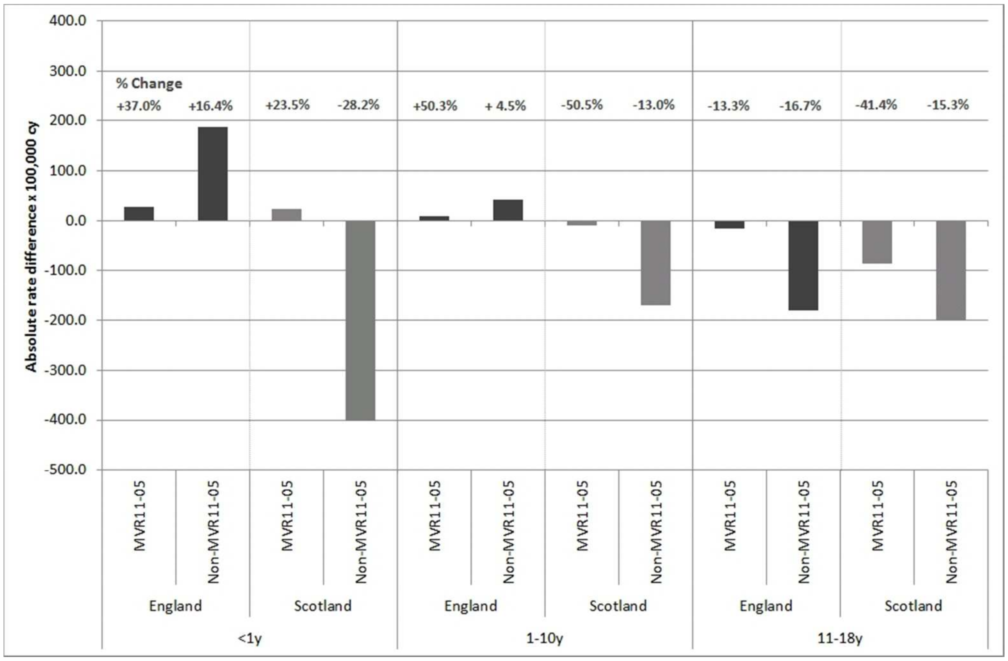

258x168mm (96 x 96 DPI)

WEB-APPENDIX

Web-Table 1 Definitions and International Classification of Diseases (ICD-10) diagnostic codes used to classify maltreatment or violence-related (MVR) injury

DEFINITIONS

Unplanned admission
An unplanned hospital admission included all longitudinally linked finished consultant episodes (FCE) classified as having an emergency or unplanned method of admission. Admissions included ordinary admissions and day cases. Transfers and injury admissions within 2 days were counted as the same event.

Injury
We classified an admission as injury if it had at least one S or T diagnostic code from the ICD-10 in any FCE

Searching strategy
We looked for injury or MVR diagnostic codes in up to 20 diagnosis fields per FCE in HES or six diagnostic fields in SMR

Exclusions
We excluded babies admitted before 7 days of age to avoid counting injuries most likely to have occurred during birth. Maternity-related admissions were also excluded.

MVR CODES AND DESCRIPTIONS	ICD-10 CODES
**Maltreatment**	
Maltreatment syndromes	T74
Effects of other deprivation (extreme neglect)	T73
Perpetrator of neglect and other maltreatment syndromes	Y06, Y07
**Assault**	
Assault by bodily force and sexual assault	Y04, Y05
Other types of assault	X85 - Y03, Y08 - Y09
**Undetermined cause**	
Events of undetermined intent	Y20 - Y34
Examination and observation following other inflicted injury	Z04.5
Examination and observation for other reasons: request for expert evidence	Z04.8
**Adverse social circumstances**	
Neonatal withdrawal symptoms from maternal use of drugs of addiction	P96.1
Other problems related to physical environment	Z58.8
Problem related to physical environment, unspecified	Z58.9
Homelessness	Z59.0
Inadequate housing	Z59.1
Lack of adequate food	Z59.4
Extreme poverty	Z59.5
Insufficient social insurance and welfare support	Z59.7
Problem related to housing and economic circumstances, unspecified	Z59.9
Problems related to social environment	Z60
Problems related to negative life events in childhood	Z61
Other problems related to upbringing	Z62
Other problems related to primary support group	Z63
Discord with counsellors	Z64.4
Problems related to other legal circumstances	Z65.3
Other specified problems related to psychosocial circumstances	Z65.8
Problem related to unspecified psychosocial circumstances	Z65.9
Problems related to lifestyle	Z72.3 - Z72.9
Problems related to care-provider dependency	Z74
Health supervision and care of foundling	Z76.1
Health supervision and care of other healthy infant and child	Z76.2
Family history of mental and behavioural disorders	Z81
Personal history of other specified risk-factors, not elsewhere classified	Z91.8

For peer review only - http://bmjopen.bmj.com/site/about/guidelines.xhtml

Web-Table 2: Change in gradient of monthly incidence trends in MVR injury in England and Scotland between 2005 and 2011 (negative binomial segmented regression analyses)

Country	Age group	1st gradient - IRR (95%CI)	Annual gradient (%)*	*p*-value for 1st gradient	change point	*p*-value for change in gradient	2nd **or** overall gradient - IRR (95%CI)	Annual gradient (%)*	*p*-value for 2nd or overall gradient
England	1w-<1y	0.998 (0.959, 1.006)	(-2.45)	0.160	November-07	0.015	1.005 (0.903, 1.010)	(6.56)	0.273
	1-10y	1.000 (0.990, 1.006)	(0.21)	0.723	December-07	0.002	1.008 (1.005, 1.011)	(10.44)	0.004
	11-18y	1.000 (0.998, 1.103)	(0.43)	0.073	July-10	<0.001	0.993 (0.947, 1.000)	(-7.54)	<0.001
Scotland	1w-<1y	0.999 (0.983, 1.089)	(-0.80)	0.860	April-09	0.057	1.014 (0.995, 1.074)	(17.98)	0.065
	1-10y					NA	0.991 (0.987, 0.994)	(-10.81)	<0.001
	11-18y	1.009 (0.999, 1.021)	(11.74)	0.003	October-06	<0.001	0.989 (0.986, 0.991)	(-12.87)	0.049

* Effective annual rate = $(IRR^{12} - 1)x100$ (calculation before rounding of IRR)

For peer review only - http://bmjopen.bmj.com/site/about/guidelines.xhtml

Web-table 3 Policy initiatives on child protection in England and Scotland 1997 – 2010 potentially having an impact on recognition and recording of child maltreatment, in healthcare

Year	England	Scotland
**1998**	The Data Protection Act 1998 **[L]**	Protecting children – A shared responsibility. Guidance for interagency co-operation **[SG]**
**1999**	Working together to safeguarding children: A guide to interagency working to safeguarding and promote the welfare of children **[SG]**	
**2000**		Protecting children – A shared responsibility. Guidance for health professionals (the pink book) **[HG]**
**2002**	The Adoption and Children Act 2002 **[L]**<br>Safeguarding children in whom illness is fabricated or induced **[SG]**	"It's everyone's job to make sure I'm alright" Report of the child protection audit e review **[S]**
**2003**	What to do if you're worried a child is being abused **[SG]**<br>Every child matters **[S]**<br>Keeping children safe: the government's response to the Victoria Climbié inquiry report and the joint chief inspectors' report Safeguarding Children **[S]**<br>Confidentiality: NHS Code of Practice **[SG]**	Getting our Priorities Right. Good Practice Guidance for working with Children and Families affected by Substance Misuse **[SG]**
**2004**	National service framework for children, young people and maternity services **[IAF]**<br>Every child matters: change for children **[S]**<br>Every child matters: next steps **[S]**<br>Children's and Maternity Services Information Strategy **[HG]**<br>Children Act 2004 **[L]**	Protecting children and young people: framework for standards **[IAF]**
**2005**	Common core of skills and knowledge for the children's workforce **[SG]**	Scottish Executive. Getting it right for every child **[S]**
**2006**	Working together to safeguarde children. **[SG]**<br>What to do if you're worried a child is being abused **[SG]**	Joint inspection of children's services and inspection of social work services (Scotland) Act 2006 **[L]**
**2007**	The 2007 Pre-budget Report and Comprehensive Spending Review **[IAF]**<br>Safeguarding children who may have been trafficked **[SG]**<br>Safeguarding children from abuse linked to a belief in spirit possession **[SG]**<br>Statutory guidance on making arrangements to safeguard and promote the welfare of children under section 11 of the Children Act 2004 **[SG]**	Protecting children and young people: Interim guidance for child protection committees for conducting a significant case review **[SG]**<br>Protection of vulnerable groups (Scotland) Act 2007 **[L]**
**2008**	The right to choose: Multi-agency statutory guidance for dealing with forced marriage **[SG]**<br>Safeguarding children in whom illness is fabricated or induced (revised) **[SG]**	A guide to implementing Getting it right for every child **[IAF]**<br><br>National domestic abuse delivery plan for children and young people **[S]**<br>The early years framework **[IAF]**

For peer review only

**2009**	NICE guidelines on when to suspect child maltreatment **[SG]**<br>Statutory guidance on promoting the health and well-being of looked after children **[SG]**<br>Safeguarding children and young people from sexual exploitation **[SG]**<br>Confidentiality: Reporting knife and gunshot wounds (supplementary guidance) **[HG]**<br>Safeguarding children: a review of arrangements in the NHS for safeguarding children **[IAF]**<br>The protection of children in England: action plan: The Government's response to Lord Laming **[S]**<br>The CQC (Registration) Regulations **[IAF]**	Sexual offences (Scotland) Act 2009 **[L]**<br>Safeguarding children in Scotland who may have been trafficked **[SG]**
**2010**	Working together to safeguarding children. A guide to interagency working to safeguarding and promote the welfare of children **[SG]**<br>NICE guidance on pregnant women with social factors **[SG]**	The national guidance for child protection in Scotland **[SG]**

Policy initiative selection criteria: We included public initiatives likely to have an impact on recognition and recording of child maltreatment, in health care. They had to be legislative acts **[L]**, statutory or inter-agency guidance **[SG]**, government strategies **[S]**, implementation or accountability frameworks **[IAF]**, or health guidance **[HG]** that applied at a national level. We excluded research or evidence synthesis and guidance published by professional bodies.

For peer review only - http://bmjopen.bmj.com/site/about/guidelines.xhtml

Web-figure 1 Monthly incidence trends from Jan 2005 to Mar 2012 of non-MVR injury in A) Infants, B) Children aged 1-10 years, and C) Adolescents aged 11-18 years, in England (dark grey**) and Scotland (**light grey**).** Faint lines represent observed rates and bold lines represent three-monthly moving averages

A)

B)

C)

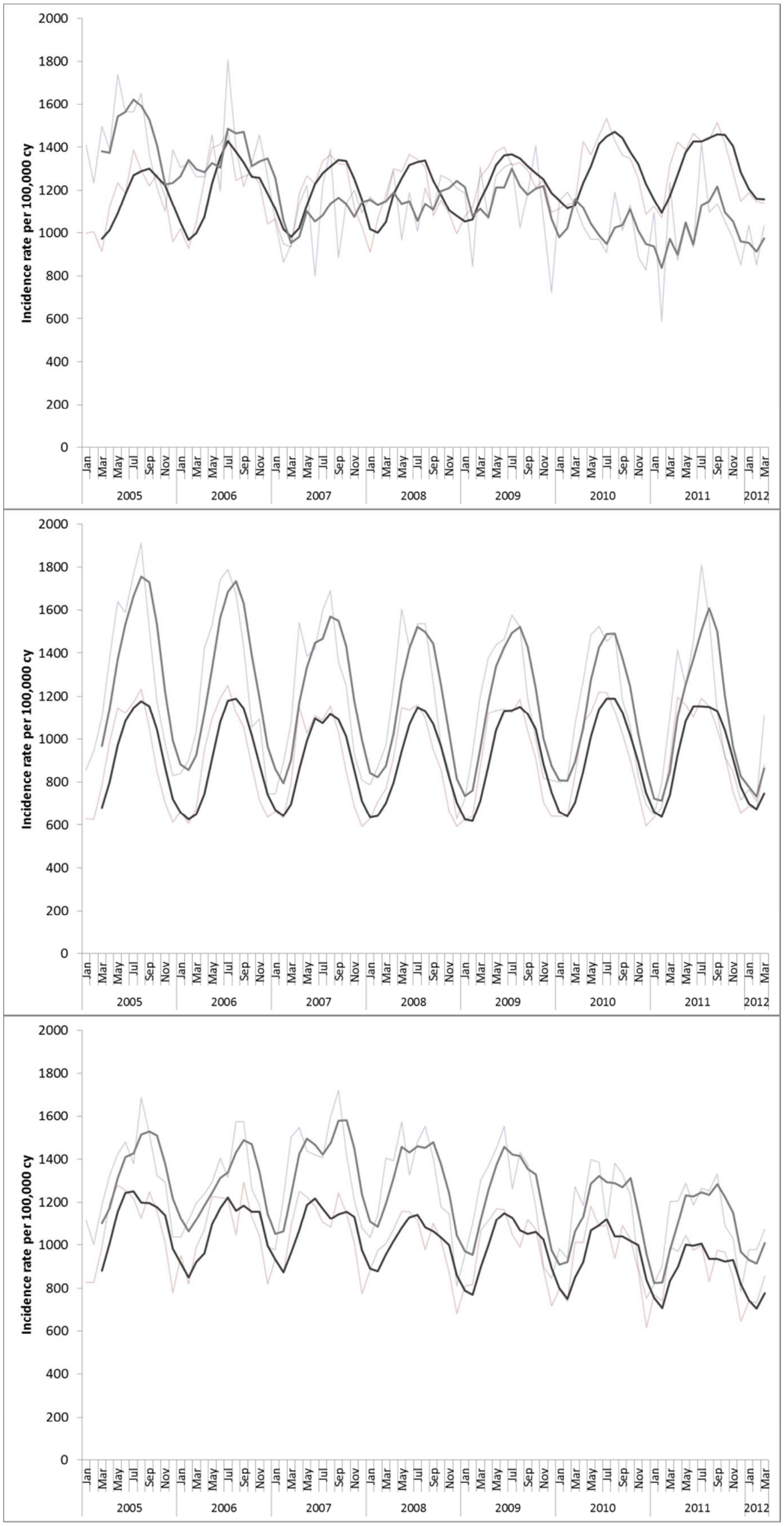

BMJ Open

Maltreatment or violence-related injury in children and adolescents admitted to the NHS: comparison of trends in England and Scotland between 2005 and 2011

Journal:	*BMJ Open*
Manuscript ID:	bmjopen-2013-004474.R1
Article Type:	Research
Date Submitted by the Author:	27-Mar-2014
Complete List of Authors:	Gonzalez-Izquierdo, Arturo; UCL Institute of Child Health, Centre of Paediatric Epidemiology and Biostatistics<br>Cortina Borja, Mario; UCL Institute of Child Health, Centre of Paediatric Epidemiology and Biostatistics<br>Woodman, Jenny; Institute of Child Health, MRC Centre of Epidemiology for Child Health<br>Mok, Jacqueline; NHS Lothian University Hospitals Division (retired),<br>McGhee, Janice; University of Edinburgh, School of Social and Political Science<br>Taylor, Julie; University of Edinburgh, Child Protection Research Centre<br>Parkin, Chloe; UCL Institute of Child Health, Centre of Paediatric Epidemiology and Biostatistics<br>Gilbert, Ruth; UCL Institute of Child Health, Centre of Paediatric Epidemiology and Biostatistics
**Primary Subject Heading**:	Public health
Secondary Subject Heading:	Health policy, Paediatrics
Keywords:	Child protection < PAEDIATRICS, Non-accidental injury < PAEDIATRICS, Health policy < HEALTH SERVICES ADMINISTRATION & MANAGEMENT

SCHOLARONE™
Manuscripts

Title: Maltreatment or violence-related injury in children and adolescents admitted to the NHS: comparison of trends in England and Scotland between 2005 and 2011

Author list:

Arturo Gonzalez-Izquierdo[1], Mario Cortina-Borja[1], Jenny Woodman[1], Jacquie Mok[2], Janice McGhee[3], Julie Taylor[4], Chloe Parkin[1], Ruth Gilbert[1]

Author affiliations:

1	Dr Arturo Gonzalez-Izquierdo, Research Associate Statistician, arturo.gonzalez-izquierdo@ucl.ac.uk<br><br>Dr Mario Cortina-Borja, Senior Lecturer in Statistics, m.cortina@ucl.ac.uk<br><br>Jenny Woodman, PhD Research Student, j.woodman@ucl.ac.uk<br><br>Dr Chloe Parkin, Senior Research Associate, c.parkin@ucl.ac.uk<br><br>Professor Ruth Gilbert, Professor of Clinical Epidemiology, r.gilbert@ucl.ac.uk<br><br>Centre of Paediatric Epidemiology and Biostatistics, UCL Institute of Child Health, 30 Guilford Street, London WC1N 1EH, UK
2	Dr Jacqueline Mok, Lead Paediatrician in Child Protection, jacqui.mok@doctors.org.uk<br><br>Retired from clinical practice, formerly NHS Lothian University Hospitals Division
3	Janice McGhee, Senior Lecturer in Social Work, Janice.McGhee@ed.ac.uk<br><br>School of Social and Political Science, the Chrystal Macmillan Building, 15a George Square, Edinburgh, EH8 9LD
4	Julie Taylor, Professor of Child Protection, julie.taylor@ed.ac.uk<br><br>Child Protection Research Centre, University of Edinburgh, St Leonard's Land, Holyrood Road, Edinburgh EH8 8AQ

Corresponding author: Professor Ruth Gilbert, MRC Centre of Epidemiology for Child Health, UCL Institute of Child Health, 30 Guilford Street, London WC1N 1EH

Telephone: 0207 905 2101, FX: 0207 905 2793, e-mail: r.gilbert@ucl.ac.uk

3,539 words

For peer review only - http://bmjopen.bmj.com/site/about/guidelines.xhtml

KEY MESSAGES

What is already known:

- Trends in violence-related injury among adolescents have been decreasing in England and Scotland
- Scotland had higher rates of violence-related injury among adolescents than England in 2005

What this study adds:

- In Scotland between 2005 and 2011, maltreatment or violence-related injury admissions increased in infants but declined steeply among children aged 1-10 and 11-18 years along with declines in other injury admissions in all age groups.
- In England, rates of maltreatment or violence-related injury admission increased in infants and 1-10 year olds along with increases in other injury admissions, and declined in adolescents, though less steeply than in Scotland.

For peer review only

For peer review only - http://bmjopen.bmj.com/site/about/guidelines.xhtml

ABSTRACT (283 words) max 300w

Objective: Legislation to safeguard children from maltreatment by carers or violence by others was advanced in England and Scotland around 2004/5 and resulted in different policies and services. We examined whether subsequent trends in injury admissions to hospital related to maltreatment or violence varied between the two countries.

Setting and participants: We analysed rates of all unplanned injury admission to National Health Service (NHS) hospitals in England and Scotland between 2005 and 2011 for children and adolescents aged less than 19 years.

Outcomes: We compared incidence trends for maltreatment or violence-related injury (MVR) and adjusted rate differences between 2005 and 2011 using Poisson or negative binomial regression models to adjust for seasonal effects and secular trends in non-MVR injury. Infants, children 1-10 years and adolescents 11-18 years were analysed separately.

Results: In 2005, MVR-rates were similar in England and Scotland for infants and 1-10 year olds, but almost twice as high in Scotland for 11-18 year olds. MVR-rates for infants increased by similar amounts in both countries, in line with rising non-MVR rates in England but contrary to declines in Scotland. Among 1-10 year olds, MVR-rates increased in England and declined in Scotland, in line with increasing non-MVR rates in England and declining rates in Scotland. Among 11-18 year olds, MVR-rates declined more steeply in Scotland than in England along with declines in non-MVR trends.

Conclusion: Diverging trends in England and Scotland may reflect true changes in the occurrence of MVR injury or differences in the way services recognise and respond to these children, record such injuries, or a combination of these factors. Further linkage of data from surveys and services for child maltreatment and violence could help distinguish the impact of policies.

For peer review only

STRENGTHS AND LIMITATIONS OF THIS STUDY

- Maltreatment or violence related injuries were analysed only if they resulted in admission to hospital. We could not determine whether children and adolescents with these injuries were instead being managed in other settings such as outpatient or emergency departments or primary care or not presenting to health care.
- Differences in coding practices, admission thresholds, performance targets, allocation of resources or service configuration could potentially influence trends.
- Strengths are that both countries have national health services and standardised, comparable hospital administrative data captured at a national level.
- A further strength is that analyses took into account background trends in admissions for other types of injuries, thereby partially accounting for admission thresholds related to system factors such as waiting times in the emergency department.

For peer review only

BACKGROUND

Exposure to maltreatment or violence is common during childhood. The term child maltreatment refers to any form of physical, emotional or sexual abuse or neglect that results in actual or potential harm to a child.[1,2] Among young children, maltreatment is mainly caused by carers. During adolescence, maltreatment more often results from violence by people other than carers, and includes assault or bullying by peers, siblings, other family members or strangers. Community surveys estimate that 40% to 60% of adolescents have been exposed to maltreatment by carers or abuse or violence by others during the previous year and around 1 in 10 of them would have been injured as a result.[3,4] Multiple types of abuse or violence often co-occur and are linked to adverse psychological outcome.[5,6]

We conducted an ecological comparison of trends in hospital admissions for maltreatment or violence-related (MVR) injury admissions in England and Scotland from 2005, when both countries implemented new legislation to safeguard children. Scotland also implemented policies from 2005 to reduce violence. We evaluated the continuum of injury related to maltreatment by carers or violence by others (e.g. peers, siblings, and strangers) for three reasons. First, clinicians dealing with injured children and adolescents may not easily distinguish the perpetrator of an inflicted injury, instead recording uncertainty about the cause or concerns about the child's environment. Second, we measured injury related to maltreatment or violence, rather than definitively caused by, as only a small minority of cases where maltreatment is suspected proceed to child protection assessment and definitive attribution of cause.[7,8] Third, policies to reduce violence may also reduce child maltreatment, and vice versa.[9,10]

Using an ecological comparison of trends in MVR injury admission to hospital, we aimed to generate hypotheses about reasons for variation between the two countries. Correlation with specific policy initiatives is difficult however, because of the variety of policy, service and societal influences.[11] Policies can impact trends in MVR injury through a variety of mechanisms. Policies to improve recognition of and responses to child maltreatment or violence may increase awareness but could also reduce occurrence. Second, policies affecting socioeconomic inequalities, social cohesion, antisocial behaviour and welfare policies to improve support for disadvantaged families, might also affect rates of maltreatment or violence.[1,12-15] Third, policies that reduce risk factors for serious injury requiring hospital admission, such as use of knives or other weapons, excessive alcohol consumption, and unregulated drug use, might reduce the rate of severe injuries requiring admission to hospital.[16,17] We discuss our findings on trends in the two countries in relation to policies to safeguard children and the wider healthcare context.

For peer review only - http://bmjopen.bmj.com/site/about/guidelines.xhtml

METHODS

We analysed trends in monthly population incidence rates of unplanned MVR injury admission to hospital between 1st of January 2005 and 31st of March 2012 in England and Scotland. We used hospital administrative data for all NHS admissions of children in England (Hospital Episode Statistics – HES) and Scotland (Scottish Morbidity Records – SMR) to identify unplanned injury admissions using previously published methods (see Web-table 1 for definitions).[18,19] We defined MVR injury using a cluster of codes from the International Classification of Diseases (ICD10) recorded in any diagnostic field at discharge (up to 20 diagnostic fields per episode in HES or six diagnostic fields in SMR).

Diagnostic coding by professional coders using case notes and discharge letters completed by clinicians is long-established and the accuracy has long been debated.[1,20,21] A recent systematic review found moderate accuracy of coding in hospital administrative data in the UK.[22] Studies using internal validation to compare clusters of ICD codes for detecting maltreatment-related injury with case notes or child protection agency data reported high specificity for clinician concerns about maltreatment,[7] and moderate specificity for definitive evidence of maltreatment or child protection agency notification.[23-26] Studies using external validation to determine whether codes in different settings produce similar rates and risk factors provide weak evidence that codes for maltreatment are measuring a similar underlying entity.[11,27]

We used previously developed maltreatment or violence-related injury (MVR) codes that were developed to be consistent with alert features mentioned in the National Institute of Health and Clinical Excellence (NICE) guidance for considering maltreatment.[11,27,28] An evaluation of this coding cluster against clinical records is reported elsewhere.[7] The cluster of codes includes four subgroups (Web-table 1). These comprise specific references to maltreatment-syndrome, assault, unexplained injury, based on codes indicating the need for further evidence to determine the intent of injury (undetermined cause), and codes reflecting concerns about the child's social circumstances, family environment and adequacy of care; factors that in combination with an injury should alert clinicians to consider the possibility of maltreatment. We used admission rather than child, as the unit of analysis as very few children (<3%) had repeat maltreatment or violence-related injury admissions within a given year (unpublished, data available from authors).

Denominator populations were derived from mid-year population estimates by year of age and calendar year published by the Office for National Statistics in England and the General Register Office for Scotland.[29,30] Analyses were stratified into three age groups reflecting broad stages of dependency, socialisation and exposure to violence (infants <1 year – non-ambulatory, children 1 to 10 years ambulatory and mixing socially under parental supervision, and adolescents 11 to 18 completed years – school age and social mixing outside parental supervision), which might be

For peer review only - http://bmjopen.bmj.com/site/about/guidelines.xhtml

amenable to different policies. Maltreatment or violence-related injury in infancy is likely to reflect abuse or neglect by carers. Between 1 and 10 years of age, maltreatment or violence-related injury can reflect abuse or neglect by carers or inadequate protection of children from abuse or neglect by others. Among 11 to 18 year olds, physical violence by carers occurs at least as frequently as at younger ages, but violence due to other family members, peers, or strangers becomes much more frequent than that perpetrated by carers.[6,31]

Analyses

Our study builds on previous reports where we have used trends in annual incidence rates in cross country comparisons for children aged 10 years or less.[11,27,28] In this study, we plotted monthly incidence rates using three-monthly moving average rates. We used time series analyses and fitted segmented-Poisson and negative binomial regression models (parametrised as generalised linear models) to determine trends and the possible timing of changes in gradient. [32-35] We took account of underlying trends in injury admission rates by adjusting analyses for unplanned injury admissions that were not related to maltreatment or violence (non-MVR). We also fitted sine and/or cosine terms to account for annual seasonal variation. Changes in the goodness-of-fit produced by including these periodic components were measured by the Akaike's information criterion (AIC). To determine whether trends significantly changed direction over time, we fitted segmented models with up to one change point. Negative binomial regression models were fitted to account for over-dispersion as the variance of MVR injury rates is likely to increase for increasing rate values. We compared goodness-of-fit across nested Poisson and negative binomial models using the log-likelihood ratio test. Details of the model are reported in Web-appendix 1.

Because of the relatively large number of parameters in the models and limited power resulting from small numbers of monthly incidence points, we analysed each country separately and report qualitative differences. In each country we estimated absolute differences between adjusted rates in calendar years 2005 and 2011 within each age group for MVR and non-MVR injury incidence rates. We also report gradients given by the change point models (and their 95% confidence intervals), and the *p*-values associated with the gradients and, where relevant, with the change in gradient during the study period. A *p*-value <0.05 was considered significant. We plotted smoothed incidence rates predicted by the model, using a non-parametric adaptive-smoother.[36] We conducted sensitivity analyses restricted to codes reflecting maltreatment syndrome or assault. Regression models and nonparametric smoothers were fitted using the R environment for statistical computing, version 2.14.2 (http://www.R-project.org).[37] We used the R packages MASS[38] and SiZer[39] to fit piecewise linear models with Poisson and negative binomial distributions.

For peer review only - http://bmjopen.bmj.com/site/about/guidelines.xhtml

RESULTS

There were 54,207 MVR injury admissions in England and 7,367 in Scotland during the study period (Jan 2005 – Mar 2012). MVR injury admission rates were distributed similarly across age groups in both countries, with the vast majority occurring in adolescents 11-18 years, (78.2% in England and 88.3%in Scotland; Table 1). The age-related incidence of MVR injury admission was J-shaped with a subsidiary peak in infancy and a major peak in late adolescence (Table 1). MVR injury admissions in the 11-18 year old age group accounted for 10% (England) to 13% (Scotland) of all unplanned injury admissions in this age group.

Figure 1 A,B,C shows MVR incidence trends in England and Scotland by age group. Negative binomial models performed significantly better in all age groups (*p*-value < 0.05); except for the analysis of rates in Scottish infants where the corresponding Poisson model is reported (Figure 1 and Web-table 2). With the exception of infants, monthly trends showed marked seasonal patterns in both countries with peaks in the spring-summer months.

For infants, the incidence of MVR injury admission increased between 2005 and 2011 in both countries: in England 27.8 and in Scotland 23.5 per 100,000 more infants were admitted for MVR injury in 2011 compared with in 2005 (Table 2, Figure 1A). This represented an increase of 37% from 2005 rates in England and of 23% in Scotland (Figure 2). The increase in England appears to have been largely determined by the background increase in non-MVR injury admissions as adjustment for the non-MVR trend and seasonal variation showed a 6.6% annual increase that was not significant at the 5% level.

In Scotland, the 18% annual increase in MVR admissions among infants estimated by the multivariable model did not reach a significant level (*p*=0.065) and was in contrast to the decline in non-MVR injury admissions (Figure 1, Web-table 2). The absolute increase between 2005 and 2011 in admission rates for non-MVR injury in England and decrease in Scotland is shown in Table 2 and Figure 2. Trends for non-MVR injury are shown in Web-figure 1A.

Among children aged 1-10 years, the incidence of MVR injury admission increased between 2005 and 2011 in England (rate difference 7.8/100,000) and declined similarly in Scotland (rate difference 9.9/100,000; Table 2, Figure 1B). This represented an increase of 50% from 2005 rates in England, and a decrease of 50% in Scotland (Figure 2). Using multivariable models to adjust for trends in non-MVR injury admissions and seasonal variation, we estimated diverging annual incidence trends: rates were increasing by 10.4% per year in England and declining by 10.8% per year in Scotland. Both these trends were significant at the 5% level (Figure 1B, Web-table 2). In this age group, admissions for non-MVR injury increased in England and decreased in Scotland (Table 2 and Web-figure 1B).

For peer review only - http://bmjopen.bmj.com/site/about/guidelines.xhtml

Among adolescents aged 11-18 years, admission rates for MVR injury in 2005 were almost twice as high in Scotland as in England (Table 2, Figure 1C). A steep decline from the autumn of 2006 in Scotland resulted in converging rates in the two countries by 2011 as rates in England declined more slowly (Figure 1C). The absolute difference in rates between 2005 and 2011 resulted in 15.8/100,000 fewer adolescents admitted with MVR injury in 2011 in England and 85.2/100,000 fewer in Scotland, relative reductions of 13.3% and 41.4% respectively (Table 2, Figure 2, Web-table 2). These trends were steeper than the declining trends in non-MVR admissions in both countries and were significant at the 5% level after adjusting for trends in non-MVR injury admissions and seasonal variation (Web-table 2). We estimated an annual decline in the incidence of MVR injury admissions in England of 7.5%, which dated from 2010. The decline for 11-18 year olds in Scotland was steeper (12.9%) and dated from 2006 (Figure 1C, Web-table 2). The rate of admission for non-MVR injury declined similarly in both countries (Figure 2, Web-figure 1C).

In sensitivity analyses that restricted MVR injury to codes for maltreatment syndrome or assault, qualitative findings were unchanged but none of the differences between England and Scotland reached significance at the 5% level (appendix - web table 1). Maltreatment syndrome or assault codes accounted for 63.1% of all childhood MVR admissions in England and 73.5% in Scotland. For infants and 1-10 year olds, we found weak evidence for increasing trends in England but small numbers in Scotland prevented modelling of trends in these age groups. For 11-18 year olds, declines using restricted MVR codes were steeper in England (12.4% annually) and similar to the decline in Scotland (12.1%).

For peer review only

DISCUSSION

Between 2005 and 2011 rates of maltreatment or violence-related injury admission increased in England among infants and 1-10 year olds along with rises in other injury admissions and declined in adolescents, though less steeply than in Scotland. Maltreatment or violence-related injury admissions in Scotland increased in infants but declined steeply among children aged 1-10 and 11-18 years along with declines in other injury admissions in all age groups. Similarities between England and Scotland were increasing rates of MVR injury admissions among infants and decreasing rates among 11-18 year olds.

Among 1-10 year olds, incidence trends for MVR injury admissions diverged between England (increasing) and Scotland (decreasing), but were consistent with trends for other injuries in this age group. Among 11-18 year olds, rates of MVR injury admission were twice as high in Scotland as in England in 2005, but fell more steeply than in England, resulting in similar rates by 2011.

Limitations of the study centre on whether these trends reflect the occurrence of MVR injury severe enough to require admission or whether they relate to differences in coding or health service thresholds for admission of children with MVR injury. First, one factor contributing to diverging rates could be improvements in the sensitivity of coding in England where coding depth is incentivised by the remuneration system 'payment by results', a system which does not operate in Scotland.[40,41]

Second, changes in admission thresholds could differentially affect rates in both countries. We confined our analyses to admissions, rather than emergency departments (ED) or primary care because coded data are not available on a national basis for non-admitted patients. However, admissions are the 'tip of the iceberg' in terms of health care attendances for MVR injury – reflecting only a minority of those presenting to the ED and primary care.[7,42,43] Flows of patients from the ED to short stay admissions may have increased following introduction of four-hour wait targets in the ED.[44] However, these targets were implemented in both Scotland and England in 2004.[45] Moreover, we adjusted trends for background changes in non-MVR injury admissions, which would have been most affected by changes to ED department waiting times.

Differential changes between countries in admission threshold specifically for MVR injuries are possible. We previously reported steep declines in maltreatment-related injury admissions in Manitoba, Canada, following a change in policy to investigate possible maltreatment in the community, avoiding admission to hospital when not medically justified.[11] We are not aware of any explicit policies to shift investigation of alleged maltreatment from the hospital to the community In England or Scotland. However, better coordination of safeguarding services in the community in Scotland compared with England, for example as a result of the 'Getting it right for every child' policy (discussed below), could potentially have contributed to declines in Scotland.

For peer review only - http://bmjopen.bmj.com/site/about/guidelines.xhtml
For peer review only

The major limitation of the study is the ecological design, which provides evidence of diverging trends but does not demonstrate which policies or practices might be associated with these different trends.

Policies related to child maltreatment or violence

To identify policies that may potentially have influenced trends in MVR injury admissions, we asked researchers in England and Scotland to independently list policy initiatives related to child maltreatment that applied nationally in each country, without knowledge of the results of the trend analyses. Relevant legislation, government strategies, inter-agency guidance, implementation or accountability frameworks or health guidance documents were included, but research evidence and guidance by professional bodies were excluded. The results, summarised in Web-table 3, show at least one set of guidance published in one or both countries every year since 2002.

Both countries saw comparable 'integrative' policies initiated during the first half of the 2000s. "Every child matters" in England and "Getting it right for every child" – GIRFEC – in Scotland, aimed to promote cooperation across sectors and agencies working for the wellbeing of children.[46] However, implementation differed between the two countries. In England, 'Every Child Matters' introduced a new structure of children's centres to coordinate targeted and specialist services, separately from existing services.[47] In contrast, GIRFEC promoted coordination within existing structures to integrate universal and targeted services.[48] It is hard to relate the timing of implementation of these two major policy initiatives to changes in trends observed in our study as implementation was gradual. For example, GIRFEC was launched in 2005 but took several years to be fully implemented.[49,50]

Management of children with suspected maltreatment or neglect within the community may have changed, particularly in Scotland. In both countries, many children with suspected maltreatment are neither admitted to hospital nor attend emergency departments. However, in Scotland closer working between health and social care professionals as a result of GIRFEC, may have resulted in a large proportion of children receiving medical assessments in out-patient clinics.[51] Many are also being seen by general practitioners.[43,52,53] Within the NHS, community paediatricians have taken on a major role as 'child abuse paediatricians' and are referred children for assessments in clinics held either within or outside the hospital setting.[51] Hence small changes in admission thresholds for injury, or for specific subgroups such as those with maltreatment or violence, could have had a substantial impact on rates of admission.

The increase in the incidence of MVR injury admissions among infants in England and Scotland may reflect raised awareness of maltreatment in infants. However, alternative explanations, such as

For peer review only - http://bmjopen.bmj.com/site/about/guidelines.xhtml

coding practices or a true increase cannot be ruled out. The increase in MVR injury admissions in England predated 2008, when two events occurred that are likely to have raised awareness of maltreatment related injuries presenting to healthcare. The first was NICE guidance '*When to suspect child maltreatment*',[54] which was mandated for hospitals in England but not in Scotland. This guidance coincided with extreme media publicity between October 2008 and August 2009 of the death of Peter Connelly in north London, a 17 month old child who died from more than 50 injuries inflicted by his parents. These events may have influenced the increase in MVR injury admissions among infants in Scotland, which dated from April 2009, and was in contrast to declines in non-MVR injury admissions among infants.

Differences in health services

The broader service context may also be relevant. Scotland spends more on the NHS than England and has more GPs per capita.[55] Approaches to configuration of services post devolution have been characterised as focussing on markets and management in England and on the medical profession and cooperation in Scotland.[56] In addition, Scotland abolished the purchaser/provider split and the idea of provider competition, and recreated organisations responsible for meeting the needs of the population and running services within defined geographical areas. This may have made it easier to integrate and coordinate services, and therefore improve quality of care along the patient pathway.[55]

External evidence for changes in trends in child maltreatment and/violence

Scotland has seen a decline in referrals to the Scottish Children's Reporter Administration over the same period as the decline in MVR injury admissions.[57] Declines in violent crime reported in police statistics have been reported in both England and Scotland, and alcohol-related admissions have also declined in Scotland.[58-61] Since 2005, Scotland has implemented intensive programmes to prevent youth violence and reduce drug and alcohol misuse, focussing on vulnerable young people.[62,63] Both England and Scotland implemented the 'challenge 25' policy in 2009 to reduce youth access to alcohol,[64] but Scotland is planning to introduce minimum pricing for alcohol – a move so far resisted in England.(http://www.alcohol-focus-scotland.org.uk/ref)

Implications

Our analyses show that the incidence of MVR injury admissions in children can change substantially over time and in opposite directions in adjacent countries with similar healthcare systems. The declines in Scotland suggest that the increasing rates observed in England are not inevitable. However, which policies, if any, have influenced these changes cannot be determined from this

For peer review only - http://bmjopen.bmj.com/site/about/guidelines.xhtml
For peer review only

study. A priority for future research is to distinguish true change in the occurrence of MVR injury needing admission from changes in coding or admission thresholds. This requires analyses of all cases of MVR injury presenting to primary care, those seen as outpatients by community paediatricians, those attending the emergency department and those admitted to hospital, to understand how children are managed within the healthcare system. Such data linkages are not yet possible due to the lack of well-coded, administrative healthcare databases across health sectors, but are a stated aim of government in England and Scotland.[65,66]

Hospitalisation for maltreatment-related injury or injury due to other forms of victimisation represents considerable suffering to the child and a major cost to the health service. These results strengthen the call by WHO to widen use administrative data to improve understanding of how policy can reduce exposure of children to injury due to violence or neglect.[67] Consideration should also be given to linking survey data of adolescent self-reported exposures to health administrative data to measure service use in children and adolescents exposed to maltreatment or violence.

For peer review only

For peer review only - http://bmjopen.bmj.com/site/about/guidelines.xhtml

Acknowledgment

AGI was supported by funding from the Department of Health Policy Research Programme through funding to the Policy Research Unit in the Health of Children, Young People and Families. The views expressed are not necessarily those of the Department. We would like to thank members of the Policy Research Unit for the health of children, young people and families: Terence Stephenson, Catherine Law, Amanda Edwards, Ruth Gilbert, Steve Morris, Helen Roberts, Catherine Shaw, Russell Viner and Miranda Wolpert. We are grateful to Andrew Woolley for commenting on the policy timeline for England.

This work is supported by awards establishing the Farr Institute of Health Informatics Research at UCLP Partners from the MRC, in partnership with Arthritis Research UK, the British Heart Foundation, Cancer Research UK, the Economic and Social Research Council, the Engineering and Physical Sciences Research Council, the National Institute of Health Research, the National Institute for Social Care and Health Research (Welsh Assembly Government), the Chief Scientist.

Authors' contributions

RG and AGI conceived the paper and the analytic plan. AGI wrote the first draft and carried out the analyses. RG wrote the final version. All authors commented on the analyses and report. RG is guarantor.

Competing interests

All authors have completed the Unified Competing Interest form at www.icmje.org/coi_disclosure.pdf (available on request from the corresponding author). AGI, MCB, JW, JM, JMG, JT, CP and RG have no competing interests that may be relevant to the submitted work.

Office (Scottish Government Health Directorates) and the Wellcome Trust (MR/K006584/1).

Data sharing

Additional data can be accessed in the web-appendix. Source data can be accessed by researchers applying to the Information Services Division Scotland or the Health and Social Care Information Centre for England.

Licence for publication

The corresponding author has the right to grant on behalf of all authors and does grant on behalf of all authors, an exclusive licence on a worldwide basis to the BMJ Publishing Group Ltd and its licensees, to permit this article (if accepted) to be published in BMJ editions and other BMJPG

For peer review only - http://bmjopen.bmj.com/site/about/guidelines.xhtml

products and to exploit all subsidiary rights, as set out in our licence (http://resources.bmj.com/bmj/authors/checklists-forms/licence-for-publication)

Transparency declaration

Ruth Gilbert on behalf of all co-authors affirms that this manuscript is an honest, accurate, and transparent account of the study being reported; that no important aspects of the study have been omitted; and that any discrepancies from the study as planned (and, if relevant, registered) have been explained.

For peer review only

For peer review only - http://bmjopen.bmj.com/site/about/guidelines.xhtml

Reference List

1. World Health Organization. Health Topics. Child maltreatment. 2013. WHO. 2013.

2. HM Government. Working together to safeguard children. A guide to interagency working to safeguard and promote the welfare of children. 2013. London, Department for Education.

3. Finkelhor D, Turner H, Ormrod R, Hamby SL: Violence, abuse, and crime exposure in a national sample of children and youth. Pediatrics 2009, 124: 1411-1423.

4. Radford L, Corral S, Bradley C, et al.. Child abuse and neglect in the UK today. 2011. London, NSPCC.

5. Finkelhor D, Ormrod RK, Turner HA: Polyvictimization and trauma in a national longitudinal cohort. Dev Psychopathol 2007, 19: 149-166.

6. Finkelhor D, Ormrod RK, Turner HA: The developmental epidemiology of childhood victimization. J Interpers Violence 2009, 24: 711-731.

7. Gonzalez-Izquierdo A, Ward A, Smith P, et al.: Notifications for child safeguarding from an acute hospital in response to presentations to healthcare by parents. Child: care health and development, 2013, Under review.

8. Flaherty EG, Sege RD, Griffith J, et al.: From suspicion of physical child abuse to reporting: primary care clinician decision-making. Pediatrics 2008, 122: 611-619.

9. Gilbert R, Kemp A, Thoburn J, et al: Recognising and responding to child maltreatment. Lancet 2009, 373: 167-180.

10. World Health Organization Regional Office for Europe. European report on preventing child maltreatment. Edited by Sethi D, Bellis M, Hughes K, Gilbert R, Mitis F, Gauden G. 2013. World Health Organization Regional Office for Europe.

11. Gilbert R, Fluke J, O'Donnell M, et al.: Child maltreatment: variation in trends and policies in six developed countries. Lancet 2012, 379: 758-772.

12. Finkelhor D, Ormrod R, Turner H, et al Pathways to poly-victimization. Child Maltreat 2009, 14: 316-329.

13. De VM, Demyttenaere K, Bruffaerts R: The relationship between adverse childhood experiences and mental health in adulthood. A systematic literature review. Tijdschr Psychiatr 2013, 55: 259-268.

14. Farrington DP, Loeber R: Epidemiology of juvenile violence. Child Adolesc Psychiatr Clin N Am 2000, 9: 733-748.

15. Murray J, Farrington DP: Risk factors for conduct disorder and delinquency: key findings from longitudinal studies. Can J Psychiatry 2010, 55: 633-642.

For peer review only - http://bmjopen.bmj.com/site/about/guidelines.xhtml

16. Norman RE, Byambaa M, De R, et al: The long-term health consequences of child physical abuse, emotional abuse, and neglect: a systematic review and meta-analysis. PLoS Med 2012, 9: e1001349.

17. Statham J: Effective services to support children in special circumstances. Child Care Health Dev 2004, 30: 589-598.

18. NHS National Services Scotland - Information Services Division. Health and Social Care Data Dictionary - SMR01 General/ Acute Impatient and Day Cases. 2012.

19. NHS.The Information Centre for Health and Social Care. HES Online. What is the data? 2012.

20. Annest JL, Fingerhut LA, Gallagher SS, et al.: Strategies to improve external cause-of-injury coding in state-based hospital discharge and emergency department data systems: recommendations of the CDC Workgroup for Improvement of External Cause-of-Injury Coding. MMWR Recomm Rep 2008, 57: 1-15.

21. Lloyd SS, Rissing JP: Physician and coding errors in patient records. JAMA 1985, 254: 1330-1336.

22. Campbell SE, Campbell MK, Grimshaw JM, et al: A systematic review of discharge coding accuracy. J Public Health Med 2001, 23: 205-211.

23. McKenzie K, Scott D, Waller G, et al: Reliability of Routinely Collected Hospital Data for Child Maltreatment Surveillance. BMC Public Health 2011, 11: 8.

24. McKenzie K, Scott D, Fraser JA, et al: Assessing the concordance of health and child protection data for maltreated and unintentionally injured children. Inj Prev 2012, 18: 50-57.

25. Schnitzer PG, Slusher PL, Kruse RL, et al: Identification of ICD codes suggestive of child maltreatment. Child Abuse Negl 2011, 35: 3-17.

26. Schnitzer PG, Slusher P, Van Tuinen M: Child maltreatment in Missouri: combining data for public health surveillance. Am J Prev Med 2004, 27: 379-384.

27. Gonzalez-Izquierdo A, Ward A, Donnell O, et al.: Cross-country comparison of victimisation-related injury admission in children and adolescents in England and Western Australia. BMC Health Serv Res 2013, 13: 260.

28. Gonzalez-Izquierdo A, Woodman J, Copley L, et al.: Variation in recording of child maltreatment in administrative records of hospital admissions for injury in England, 1997-2009. Arch Dis Child 2010, 95: 918-925.

29. General Register Office for Scotland. Mid-Year Population Estimates. 2012.

30. Office for National Statistics. Population Estimates for UK, England and Wales, Scotland and Northern Ireland. 2012.

31. Radford L, Corral S, Bradley C, et al.. Child cruelty in the UK: An NSPCC study into child abuse and neglect over the past 30 years. 2011. London, NSPCC.

For peer review only - http://bmjopen.bmj.com/site/about/guidelines.xhtml

32. Biglan A, Ary D, Wagenaar AC: The Value of Interrupted Time-Series Experiments for Community Intervention Research. Prevention Science 2000, 1: 31-49.

33. Toms JD, Lesperance ML: Piecewise regression: a tool for identifying ecological thresholds. Ecology 2003, 84: 2034-2041.

34. Wagner AK, Soumerai SB, Zhang F, et al: Segmented regression analysis of interrupted time series studies in medication use research. Journal of Clinical Pharmacy and Therapeutics 2002, 299-309.

35. Hilbe JM: Negative binomial regression, Second Edition edn. Cambridge: Cambridge University Press; 2011.

36. Friedman JH. A variable span scatterplot smoother. Edited by Laboratory for Computational Statistics. 5. 1984. Standford University. Technical Report No. 5.

37. R Development Core Team. R: A language and environment for statistical computing. 2008. Vienna, R Foundation for Statistical Computing.

38. Venables WN, Ripley BD: Modern Applied Statistics with S, Fourth Edition edn. Springer; 2002.

39. Sonderegger DL, Wang HN, Clements WH, et al: Using SiZer to detect thresholds in ecological data. Frontiers in Ecology and Environment 2009, 7: 190-195.

40. Audit Commission. Payment by results assurance framework. 2006. Audit Commission.

41. Jameson S, Reed MR: Payment by results and coding practice in the National Health Service. The importance for orthopaedic surgeons. J Bone Joint Surg Br 2007, 89: 1427-1430.

42. Woodman J, Pitt M, Wentz R, et al: Performance of screening tests for child physical abuse in Accident and Emergency Departments. Health Technol Assess 2008, 12: iii, xi-xiii 1-95.

43. Woodman J, Freemantle N, Allister J, et al: Variation in recorded child maltreatment concerns in UK primary care records: a cohort study using The Health Improvement Network (THIN) database. PLoS One 2012, 7: e49808.

44. Gill PJ, Goldacre MJ, Mant D, et al.: Increase in emergency admissions to hospital for children aged under 15 in England, 1999-2010: national database analysis. Arch Dis Child 2013, 98: 328-334.

45. Appleby J: Are accident and emergency attendances increasing? BMJ 2013, 346: f3677.

46. Stafford A, Parton N, Vincent S, et al: Child protection systems in the United Kingdom. A comparative analysis. London, UK: Jessica Kingsley Publishers; 2012.

47. Department for Education. Every child matters. Chapter 5 Accountability and integration. 2003. The Stationary Office.

48. The Scottish Goverment. A guide to Getting it right for every child. Chapter 2 Getting it right in practice. 2012. The Scottish Goverment.

For peer review only - http://bmjopen.bmj.com/site/about/guidelines.xhtml

49. Children's Hearings System. Getting it right for every child. Proposals for action. 2005. Scottish Executive.

50. The Scottish Government. Getting it right for children and families. A guide to getting it right for every child. 2012.

51. Kirk CB, Lucas-Herald A, Mok J: Child protection medical assessments: why do we do them? Arch Dis Child 2010, 95: 336-340.

52. Woodman J, Allister J, Rafi I, et al.: A simple approach to improve recording of concerns about child maltreatment in primary care records: developing a quality improvement intervention. Br J Gen Pract 2012, 62: e478-e486.

53. Woodman J, Gilbert RE, Allister J, et al: Responses to concerns about child maltreatmentt: a qualitative study of GPs in England. BMJ Open 2013, 3, 12. e003894. doi: 10.1136/bmjopen-2013-003894.

54. National Collaborating Centre for Women's and Children's Health. When to suspect child maltreatment . 2009. London, National Institute for Health and Clinical Excellence.

55. Connolly S, Bevan G, Mays N. Funding and performance of healthcare systems in the four UK countries before and after devolution. 2010. London, The Nuffield Trust.

56. Greer S. Four Way Bet: How devolution has led to four different models for the NHS. 2004. London, The Constitution Unit.

57. McGhee J: Children's Hearings in Scotland: Balancing Rights and Welfare. In Social Work and the Law in Scotland. 2nd edition edition. Edited by Davies R, Gordon J. Milton Keynes: The Open University and Basingstoke: Palgrave Macmillan; 2011:85-100.

58. Information Services Division Scotland. Alcohol-related hospital statistics Scotland 2012/13. 2012. NHS Scotland.

59. Office for National Statistics. Crime in England and Wales. 2014.

60. The Scottish Government. High level summary of statistics trend. Crime victimisation. 2011.

61. The Scottish Government. 2010/11 Scottish Crime and Justice Survey: Main findings. 2011.

62. The Scottish Government. Youth Justice. 2013. The Scottish Goverment. 2013.

63. Gov.UK. Youth crime prevention programmes. 2013. Gov.UK. 2013.

For peer review only - http://bmjopen.bmj.com/site/about/guidelines.xhtml

64. Drink Aware. Buying alcohol. 2013. Drink Aware.

65. Health and Social Care Information Centre. Data linkage and extract service. 2014. NHS.

66. Information Services Division Scotland. Electronic data research and innovation service (eDRIS). 2013. NHS Scotland.

67. WHO. World Report on Violence and health. 1-372. 2002.

For peer review only

For peer review only - http://bmjopen.bmj.com/site/about/guidelines.xhtml

Table 1 Hospital admissions for maltreatment or violence-related (MVR) injury in children in England and Scotland between 2005 and 2011, inclusive

Country		Age group	Total unplanned injury	Unplanned injury rates per 100,000 cy *	MVR injury	MVR incidence rates per 100,000cy *	% of total MVR injury**	% of total injury ***
England		<1y	61,987	1361.3	3,955	86.9	7.3	6.4
		1-10y	402,334	962.5	7,876	18.8	14.5	2.0
		11-18y	414,259	1157.4	42,376	118.4	78.2	10.2
	Subtotal		878,580	1069.5	54,207	66.0	100	6.2
Scotland		<1y	5,168	1277.9	290	71.7	3.9	5.6
		1-10y	48,357	1244.3	570	14.7	7.7	1.2
		11-18y	51,339	1471.0	6,507	186.4	88.4	12.7
	Subtotal		104,864	1347.7	7,367	94.7	100	7.0

* Denominators are the mid-year population estimates from Office for National Statistics and General Register Office for Scotland, cy = child years
** Denominator is the total number of maltreatment or violence-related injury admissions in children 0 to 18 years of age by country
*** Denominator is the total number of unplanned injury admissions within age group & country

For peer review only

Table 2 Observed mean incidence rate per 100,000 children in calendar years 2005 and 2011 and absolute difference in rates

		Rate (95%CI) **2005**		Rate (95%CI) **2011**		Absolute difference in rates (95%CI)	
Country	Age group	MVR	Non-MVR	MVR	Non-MVR	MVR	Non-MVR
England	<1y	75.0 (68.1, 81.9)	1146.9 (1120.0, 1173.9)	102.8 (95.2, 110.4)	1334.7 (1307.2, 1362.2)	27.8 (27.1, 28.5)	187.8 (187.2, 188.3)
	1-10y	15.5 (14.5, 16.5)	909.6 (901.9, 917.3)	23.3 (22.1, 24.5)	950.6 (942.9, 958.2)	7.8 (7.6, 8.0)	41.0 (41.0, 40.9)
	11-18y	119.1 (116.2, 122.1)	1076.5 (1067.6, 1085.4)	103.3 (100.6, 106.1)	896.2 (888, 904.5)	-15.8 (-15.6, -16.0)	-180.3 (-179.6, -180.9)
Scotland	<1y	66.1 (44.5, 87.7)	1426.3 (1326.0, 1526.6)	89.6 (65.5, 113.7)	102 4.1 (942.5, 1105.6)	23.5 (21.0, 26.0)	-402.3 (-383.5, -421.0)
	1-10y	19.6 (16.0, 23.3)	1306.2 (1276.2, 1336.3)	9.7 (7.2, 12.3)	1136.6 (1108.8, 1164.3)	-9.9 (-8.8, -11.0)	-169.7 (-167.4, -172.0)
	11-18y	205.8 (193.4, 218.2)	1314.3 (1283.0, 1345.7)	120.6 (110.8, 130.5)	1113.7 (1083.8, 1143.7)	-85.2 (-82.6, -87.7)	-200.6 (-199.2, -202.0)

For peer review only

For peer review only - http://bmjopen.bmj.com/site/about/guidelines.xhtml

Figure 1 Monthly incidence trends from Jan 2005 to Mar 2012 of MVR injury in A) Infants, B) Children aged 1-10 years, and C) Adolescents aged 11-18 years, in England (dark grey**) and Scotland (**light grey**).** Faint lines represent observed rates and bold lines represent three-monthly moving averages. Dashed lines represent smoothed trends of incidence rates estimated from the segmented regression analysis (except for trends in Scottish 1-10 year-olds where a standard Poisson regression was used) and markers indicate the change point estimated by the segmented regression model.

Figure 2 Absolute rate difference between annual incidence rates in calendar years 2005 and 2011 for MVR injury and non-MVR injury admissions of children and adolescents by age group and country.

Footnote for the figure: % change reflects proportionate change measured as (absolute difference between rate in 2005 and 2011)/(rate in 2005); MVR11-05 = difference in maltreatment or violence related injury admissions between 2005 and 2011 (similarly for non-MVR11-05)

For peer review only

For peer review only - http://bmjopen.bmj.com/site/about/guidelines.xhtml

Title: Maltreatment or violence-related injury in children and adolescents admitted to the NHS: comparison of trends in England and Scotland between 2005 and 2011

Author list:

Arturo Gonzalez-Izquierdo[1], Mario Cortina-Borja[1], Jenny Woodman[1], Jacquie Mok[2], Janice McGhee[3], Julie Taylor[4], Chloe Parkin[1], Ruth Gilbert[1]

Author affiliations:

1	Dr Arturo Gonzalez-Izquierdo, Research Associate Statistician, arturo.gonzalez-izquierdo@ucl.ac.uk<br>Dr Mario Cortina-Borja, Senior Lecturer in Statistics, m.cortina@ucl.ac.uk<br>Jenny Woodman, PhD Research Student, j.woodman@ucl.ac.uk<br>Dr Chloe Parkin, Senior Research Associate, c.parkin@ucl.ac.uk<br>Professor Ruth Gilbert, Professor of Clinical Epidemiology, r.gilbert@ucl.ac.uk<br>Centre of Paediatric Epidemiology and Biostatistics, UCL Institute of Child Health, 30 Guilford Street, London WC1N 1EH, UK
2	Dr Jacqueline Mok, Lead Paediatrician in Child Protection, jacqui.mok@doctors.org.uk<br>Retired from clinical practice, formerly NHS Lothian University Hospitals Division
3	Janice McGhee, Senior Lecturer in Social Work, Janice.McGhee@ed.ac.uk<br>School of Social and Political Science, the Chrystal Macmillan Building, 15a George Square, Edinburgh, EH8 9LD
4	Julie Taylor, Professor of Child Protection, julie.taylor@ed.ac.uk<br>Child Protection Research Centre, University of Edinburgh, St Leonard's Land, Holyrood Road, Edinburgh EH8 8AQ

Corresponding author: Professor Ruth Gilbert, MRC Centre of Epidemiology for Child Health, UCL Institute of Child Health, 30 Guilford Street, London WC1N 1EH

Telephone: 0207 905 2101, FX: 0207 905 2793, e-mail: r.gilbert@ucl.ac.uk

3,539 words

For peer review only - http://bmjopen.bmj.com/site/about/guidelines.xhtml

KEY MESSAGES

What is already known:

- Trends in violence-related injury among adolescents have been decreasing in England and Scotland
- Scotland had higher rates of violence-related injury among adolescents than England in 2005

What this study adds:

- In Scotland between 2005 and 2011, maltreatment or violence-related injury admissions increased in infants but declined steeply among children aged 1-10 and 11-18 years along with declines in other injury admissions in all age groups.
- In England, rates of maltreatment or violence-related injury admission increased in infants and 1-10 year olds along with increases in other injury admissions, and declined in adolescents, though less steeply than in Scotland.

For peer review only

For peer review only - http://bmjopen.bmj.com/site/about/guidelines.xhtml

ABSTRACT (283 words) max 300w

Objective: Legislation to safeguard children from maltreatment by carers or violence by others was advanced in England and Scotland around 2004/5 and resulted in different policies and services. We examined whether subsequent trends in injury admissions to hospital related to maltreatment or violence varied between the two countries.

Setting and participants: We analysed rates of all unplanned injury admission to National Health Service (NHS) hospitals in England and Scotland between 2005 and 2011 for children and adolescents aged less than 19 years.

Outcomes: We compared incidence trends for maltreatment or violence-related injury (MVR) and adjusted rate differences between 2005 and 2011 using Poisson or negative binomial regression models to adjust for seasonal effects and secular trends in non-MVR injury. Infants, children 1-10 years and adolescents 11-18 years were analysed separately.

Results In 2005, MVR-rates were similar in England and Scotland for infants and 1-10 year olds, but almost twice as high in Scotland for 11-18 year olds. MVR-rates for infants increased by similar amounts in both countries, in line with rising non-MVR rates in England but contrary to declines in Scotland. Among 1-10 year olds, MVR-rates increased in England and declined in Scotland, in line with increasing non-MVR rates in England and declining rates in Scotland. Among 11-18 year olds, MVR-rates declined more steeply in Scotland than in England along with declines in non-MVR trends.

Conclusion Diverging trends in England and Scotland may reflect true changes in the occurrence of MVR injury or differences in the way services recognise and respond to these children, record such injuries, or a combination of these factors. Further linkage of data from surveys and services for child maltreatment and violence could help distinguish the impact of policies.

For peer review only - http://bmjopen.bmj.com/site/about/guidelines.xhtml

STRENGTHS AND LIMITATIONS OF THIS STUDY

- Maltreatment or violence related injuries were analysed only if they resulted in admission to hospital. We could not determine whether children and adolescents with these injuries were instead being managed in other settings such as outpatient or emergency departments or primary care or not presenting to health care.
- Differences in coding practices, admission thresholds, performance targets, allocation of resources or service configuration could potentially influence trends.
- Strengths are that both countries have national health services and standardised, comparable hospital administrative data captured at a national level.
- A further strength is that analyses took into account background trends in admissions for other types of injuries, thereby partially accounting for admission thresholds related to system factors such as waiting times in the emergency department.

For peer review only - http://bmjopen.bmj.com/site/about/guidelines.xhtml

BACKGROUND

Exposure to maltreatment or violence is common during childhood. The term child maltreatment refers to any form of physical, emotional or sexual abuse or neglect that results in actual or potential harm to a child.[1,2] Among young children, maltreatment is mainly caused by carers. During adolescence, maltreatment more often results from violence by people other than carers, and includes assault or bullying by peers, siblings, other family members or strangers. Community surveys estimate that 40% to 60% of adolescents have been exposed to maltreatment by carers or abuse or violence by others during the previous year and around 1 in 10 of them would have been injured as a result.[3,4] Multiple types of abuse or violence often co-occur and are linked to adverse psychological outcome.[5,6]

We conducted an ecological comparison of trends in hospital admissions for maltreatment or violence-related (MVR) injury admissions in England and Scotland from 2005, when both countries implemented new legislation to safeguard children. Scotland also implemented policies from 2005 to reduce violence. We evaluated the continuum of injury related to maltreatment by carers or violence by others (e.g. peers, siblings, and strangers) for three reasons. First, clinicians dealing with injured children and adolescents may not easily distinguish the perpetrator of an inflicted injury, instead recording uncertainty about the cause or concerns about the child's environment. Second, we measured injury related to maltreatment or violence, rather than definitively caused by, as only a small minority of cases where maltreatment is suspected proceed to child protection assessment and definitive attribution of cause.[7,8] Third, policies to reduce violence may also reduce child maltreatment, and vice versa.[9,10]

Using an ecological comparison of trends in MVR injury admission to hospital, we aimed to generate hypotheses about reasons for variation between the two countries. Correlation with specific policy initiatives is difficult however, because of the variety of policy, service and societal influences.[11] Policies can impact trends in MVR injury through a variety of mechanisms. Policies to improve recognition of and responses to child maltreatment or violence may increase awareness but could also reduce occurrence. Second, policies affecting socioeconomic inequalities, social cohesion, antisocial behaviour and welfare policies to improve support for disadvantaged families, might also affect rates of maltreatment or violence.[1,12-15] Third, policies that reduce risk factors for serious injury requiring hospital admission, such as use of knives or other weapons, excessive alcohol consumption, and unregulated drug use, might reduce the rate of severe injuries requiring admission to hospital.[16,17] We discuss our findings on trends in the two countries in relation to policies to safeguard children and the wider healthcare context.

For peer review only - http://bmjopen.bmj.com/site/about/guidelines.xhtml

METHODS

We analysed trends in monthly population incidence rates of unplanned MVR injury admission to hospital between 1st of January 2005 and 31st of March 2012 in England and Scotland. We used hospital administrative data for all NHS admissions of children in England (Hospital Episode Statistics – HES) and Scotland (Scottish Morbidity Records – SMR) to identify unplanned injury admissions using previously published methods (see Web-table 1 for definitions).[18,19] We defined MVR injury using a cluster of codes from the International Classification of Diseases (ICD10) recorded in any diagnostic field at discharge (up to 20 diagnostic fields per episode in HES or six diagnostic fields in SMR).

Diagnostic coding by professional coders using case notes and discharge letters completed by clinicians is long-established and the accuracy has long been debated.[1,20,21] A recent systematic review found moderate accuracy of coding in hospital administrative data in the UK.[22] Studies using internal validation to compare clusters of ICD codes for detecting maltreatment-related injury with case notes or child protection agency data reported high specificity for clinician concerns about maltreatment,[7] and moderate specificity for definitive evidence of maltreatment or child protection agency notification.[23-26] Studies using external validation to determine whether codes in different settings produce similar rates and risk factors provide weak evidence that codes for maltreatment are measuring a similar underlying entity.[11,27]

We used previously developed maltreatment or violence-related injury (MVR) codes that were developed to be consistent with alert features mentioned in the National Institute of Health and Clinical Excellence (NICE) guidance for considering maltreatment.[11,27,28] An evaluation of this coding cluster against clinical records is reported elsewhere.[7] The cluster of codes includes four subgroups (Web-table 1). These comprise specific references to maltreatment-syndrome, assault, unexplained injury, based on codes indicating the need for further evidence to determine the intent of injury (undetermined cause), and codes reflecting concerns about the child's social circumstances, family environment and adequacy of care; factors that in combination with an injury should alert clinicians to consider the possibility of maltreatment. We used admission rather than child, as the unit of analysis as very few children (<3%) had repeat maltreatment or violence-related injury admissions within a given year (unpublished, data available from authors).

Denominator populations were derived from mid-year population estimates by year of age and calendar year published by the Office for National Statistics in England and the General Register Office for Scotland.[29,30] Analyses were stratified into three age groups reflecting broad stages of dependency, socialisation and exposure to violence (infants <1 year – non-ambulatory, children 1 to 10 years ambulatory and mixing socially under parental supervision, and adolescents 11 to 18 completed years – school age and social mixing outside parental supervision), which might be

For peer review only - http://bmjopen.bmj.com/site/about/guidelines.xhtml

amenable to different policies. Maltreatment or violence-related injury in infancy is likely to reflect abuse or neglect by carers. Between 1 and 10 years of age, maltreatment or violence-related injury can reflect abuse or neglect by carers or inadequate protection of children from abuse or neglect by others. Among 11 to 18 year olds, physical violence by carers occurs at least as frequently as at younger ages, but violence due to other family members, peers, or strangers becomes much more frequent than that perpetrated by carers.[6,31]

Analyses

Our study builds on previous reports where we have used trends in annual incidence rates in cross country comparisons for children aged 10 years or less.[11,27,28] In this study, we plotted monthly incidence rates using three-monthly moving average rates. We used time series analyses and fitted segmented-Poisson and negative binomial regression models (parametrised as generalised linear models) to determine trends and the possible timing of changes in gradient. [32-35] We took account of underlying trends in injury admission rates by adjusting analyses for unplanned injury admissions that were not related to maltreatment or violence (non-MVR). We also fitted sine and/or cosine terms to account for annual seasonal variation. Changes in the goodness-of-fit produced by including these periodic components were measured by the Akaike's information criterion (AIC). To determine whether trends significantly changed direction over time, we fitted segmented models with up to one change point. Negative binomial regression models were fitted to account for over-dispersion as the variance of MVR injury rates is likely to increase for increasing rate values. We compared goodness-of-fit across nested Poisson and negative binomial models using the log-likelihood ratio test. Details of the model are reported in Web-appendix 1.

Because of the relatively large number of parameters in the models and limited power resulting from small numbers of monthly incidence points, we analysed each country separately and report qualitative differences. In each country we estimated absolute differences between adjusted rates in calendar years 2005 and 2011 within each age group for MVR and non-MVR injury incidence rates. We also report gradients given by the change point models (and their 95% confidence intervals), and the *p*-values associated with the gradients and, where relevant, with the change in gradient during the study period. A *p*-value <0.05 was considered significant. We plotted smoothed incidence rates predicted by the model, using a non-parametric adaptive-smoother.[36] We conducted sensitivity analyses restricted to codes reflecting maltreatment syndrome or assault. Regression models and nonparametric smoothers were fitted using the R environment for statistical computing, version 2.14.2 (http://www.R-project.org).[37] We used the R packages MASS[38] and SiZer[39] to fit piecewise linear models with Poisson and negative binomial distributions.

For peer review only - http://bmjopen.bmj.com/site/about/guidelines.xhtml

RESULTS

There were 54,207 MVR injury admissions in England and 7,367 in Scotland during the study period (Jan 2005 – Mar 2012). MVR injury admission rates were distributed similarly across age groups in both countries, with the vast majority occurring in adolescents 11-18 years, (78.2% in England and 88.3%in Scotland; Table 1). The age-related incidence of MVR injury admission was J-shaped with a subsidiary peak in infancy and a major peak in late adolescence (Table 1). MVR injury admissions in the 11-18 year old age group accounted for 10% (England) to 13% (Scotland) of all unplanned injury admissions in this age group.

Figure 1 A,B,C shows MVR incidence trends in England and Scotland by age group. Negative binomial models performed significantly better in all age groups (*p*-value < 0.05); except for the analysis of rates in Scottish infants where the corresponding Poisson model is reported (Figure 1 and Web-table 2). With the exception of infants, monthly trends showed marked seasonal patterns in both countries with peaks in the spring-summer months.

For infants, the incidence of MVR injury admission increased between 2005 and 2011 in both countries: in England 27.8 and in Scotland 23.5 per 100,000 more infants were admitted for MVR injury in 2011 compared with in 2005 (Table 2, Figure 1A). This represented an increase of 37% from 2005 rates in England and of 23% in Scotland (Figure 2). The increase in England appears to have been largely determined by the background increase in non-MVR injury admissions as adjustment for the non-MVR trend and seasonal variation showed a 6.6% annual increase that was not significant at the 5% level.

In Scotland, the 18% annual increase in MVR admissions among infants estimated by the multivariable model did not reach a significant level (*p*=0.065) and was in contrast to the decline in non-MVR injury admissions (Figure 1, Web-table 2). The absolute increase between 2005 and 2011 in admission rates for non-MVR injury in England and decrease in Scotland is shown in Table 2 and Figure 2. Trends for non-MVR injury are shown in Web-figure 1A.

Among children aged 1-10 years, the incidence of MVR injury admission increased between 2005 and 2011 in England (rate difference 7.8/100,000) and declined similarly in Scotland (rate difference 9.9/100,000; Table 2, Figure 1B). This represented an increase of 50% from 2005 rates in England, and a decrease of 50% in Scotland (Figure 2). Using multivariable models to adjust for trends in non-MVR injury admissions and seasonal variation, we estimated diverging annual incidence trends: rates were increasing by 10.4% per year in England and declining by 10.8% per year in Scotland. Both these trends were significant at the 5% level (Figure 1B, Web-table 2). In this age group, admissions for non-MVR injury increased in England and decreased in Scotland (Table 2 and Web-figure 1B).

For peer review only - http://bmjopen.bmj.com/site/about/guidelines.xhtml

Among adolescents aged 11-18 years, admission rates for MVR injury in 2005 were almost twice as high in Scotland as in England (Table 2, Figure 1C). A steep decline from the autumn of 2006 in Scotland resulted in converging rates in the two countries by 2011 as rates in England declined more slowly (Figure 1C). The absolute difference in rates between 2005 and 2011 resulted in 15.8/100,000 fewer adolescents admitted with MVR injury in 2011 in England and 85.2/100,000 fewer in Scotland, relative reductions of 13.3% and 41.4% respectively (Table 2, Figure 2, Web-table 2). These trends were steeper than the declining trends in non-MVR admissions in both countries and were significant at the 5% level after adjusting for trends in non-MVR injury admissions and seasonal variation (Web-table 2). We estimated an annual decline in the incidence of MVR injury admissions in England of 7.5%, which dated from 2010. The decline for 11-18 year olds in Scotland was steeper (12.9%) and dated from 2006 (Figure 1C, Web-table 2). The rate of admission for non-MVR injury declined similarly in both countries (Figure 2, Web-figure 1C).

In sensitivity analyses that restricted MVR injury to codes for maltreatment syndrome or assault, qualitative findings were unchanged but none of the differences between England and Scotland reached significance at the 5% level (appendix - web table 1). Maltreatment syndrome or assault codes accounted for 63.1% of all childhood MVR admissions in England and 73.5% in Scotland. For infants and 1-10 year olds, we found weak evidence for increasing trends in England but small numbers in Scotland prevented modelling of trends in these age groups. For 11-18 year olds, declines using restricted MVR codes were steeper in England (12.4% annually) and similar to the decline in Scotland (12.1%).

For peer review only - http://bmjopen.bmj.com/site/about/guidelines.xhtml

DISCUSSION

Between 2005 and 2011 rates of maltreatment or violence-related injury admission increased in England among infants and 1-10 year olds along with rises in other injury admissions and declined in adolescents, though less steeply than in Scotland. Maltreatment or violence-related injury admissions in Scotland increased in infants but declined steeply among children aged 1-10 and 11-18 years along with declines in other injury admissions in all age groups. Similarities between England and Scotland were increasing rates of MVR injury admissions among infants and decreasing rates among 11-18 year olds.

Among 1-10 year olds, incidence trends for MVR injury admissions diverged between England (increasing) and Scotland (decreasing), but were consistent with trends for other injuries in this age group. Among 11-18 year olds, rates of MVR injury admission were twice as high in Scotland as in England in 2005, but fell more steeply than in England, resulting in similar rates by 2011.

Limitations of the study centre on whether these trends reflect the occurrence of MVR injury severe enough to require admission or whether they relate to differences in coding or health service thresholds for admission of children with MVR injury. First, one factor contributing to diverging rates could be improvements in the sensitivity of coding in England where coding depth is incentivised by the remuneration system ‘payment by results’, a system which does not operate in Scotland.[40,41]

Second, changes in admission thresholds could differentially affect rates in both countries. We confined our analyses to admissions, rather than emergency departments (ED) or primary care because coded data are not available on a national basis for non-admitted patients. However, admissions are the ‘tip of the iceberg’ in terms of health care attendances for MVR injury – reflecting only a minority of those presenting to the ED and primary care.[7,42,43] Flows of patients from the ED to short stay admissions may have increased following introduction of four-hour wait targets in the ED.[44] However, these targets were implemented in both Scotland and England in 2004.[45] Moreover, we adjusted trends for background changes in non-MVR injury admissions, which would have been most affected by changes to ED department waiting times.

Differential changes between countries in admission threshold specifically for MVR injuries are possible. We previously reported steep declines in maltreatment-related injury admissions in Manitoba, Canada, following a change in policy to investigate possible maltreatment in the community, avoiding admission to hospital when not medically justified.[11] We are not aware of any explicit policies to shift investigation of alleged maltreatment from the hospital to the community In England or Scotland. However, better coordination of safeguarding services in the community in Scotland compared with England, for example as a result of the ‘Getting it right for every child’ policy (discussed below), could potentially have contributed to declines in Scotland.

For peer review only - http://bmjopen.bmj.com/site/about/guidelines.xhtml

The major limitation of the study is the ecological design, which provides evidence of diverging trends but does not demonstrate which policies or practices might be associated with these different trends.

Policies related to child maltreatment or violence

To identify policies that may potentially have influenced trends in MVR injury admissions, we asked researchers in England and Scotland to independently list policy initiatives related to child maltreatment that applied nationally in each country, without knowledge of the results of the trend analyses. Relevant legislation, government strategies, inter-agency guidance, implementation or accountability frameworks or health guidance documents were included, but research evidence and guidance by professional bodies were excluded. The results, summarised in Web-table 3, show at least one set of guidance published in one or both countries every year since 2002.

Both countries saw comparable ‘integrative’ policies initiated during the first half of the 2000s. “Every child matters” in England and “Getting it right for every child” – GIRFEC – in Scotland, aimed to promote cooperation across sectors and agencies working for the wellbeing of children.[46] However, implementation differed between the two countries. In England, ‘Every Child Matters’ introduced a new structure of children’s centres to coordinate targeted and specialist services, separately from existing services.[47] In contrast, GIRFEC promoted coordination within existing structures to integrate universal and targeted services.[48] It is hard to relate the timing of implementation of these two major policy initiatives to changes in trends observed in our study as implementation was gradual. For example, GIRFEC was launched in 2005 but took several years to be fully implemented.[49,50]

Management of children with suspected maltreatment or neglect within the community may have changed, particularly in Scotland. In both countries, many children with suspected maltreatment are neither admitted to hospital nor attend emergency departments. However, in Scotland closer working between health and social care professionals as a result of GIRFEC, may have resulted in a large proportion of children receiving medical assessments in out-patient clinics.[51] Many are also being seen by general practitioners.[43,52,53] Within the NHS, community paediatricians have taken on a major role as ‘child abuse paediatricians’ and are referred children for assessments in clinics held either within or outside the hospital setting.[51] Hence small changes in admission thresholds for injury, or for specific subgroups such as those with maltreatment or violence, could have had a substantial impact on rates of admission.

The increase in the incidence of MVR injury admissions among infants in England and Scotland may reflect raised awareness of maltreatment in infants. However, alternative explanations, such as

For peer review only - http://bmjopen.bmj.com/site/about/guidelines.xhtml

coding practices or a true increase cannot be ruled out. The increase in MVR injury admissions in England predated 2008, when two events occurred that are likely to have raised awareness of maltreatment related injuries presenting to healthcare. The first was NICE guidance '*When to suspect child maltreatment*',[54] which was mandated for hospitals in England but not in Scotland. This guidance coincided with extreme media publicity between October 2008 and August 2009 of the death of Peter Connelly in north London, a 17 month old child who died from more than 50 injuries inflicted by his parents. These events may have influenced the increase in MVR injury admissions among infants in Scotland, which dated from April 2009, and was in contrast to declines in non-MVR injury admissions among infants.

Differences in health services

The broader service context may also be relevant. Scotland spends more on the NHS than England and has more GPs per capita.[55] Approaches to configuration of services post devolution have been characterised as focussing on markets and management in England and on the medical profession and cooperation in Scotland.[56] In addition, Scotland abolished the purchaser/provider split and the idea of provider competition, and recreated organisations responsible for meeting the needs of the population and running services within defined geographical areas. This may have made it easier to integrate and coordinate services, and therefore improve quality of care along the patient pathway.[55]

External evidence for changes in trends in child maltreatment and/violence

Scotland has seen a decline in referrals to the Scottish Children's Reporter Administration over the same period as the decline in MVR injury admissions.[57] Declines in violent crime reported in police statistics have been reported in both England and Scotland, and alcohol-related admissions have also declined in Scotland.[58-61] Since 2005, Scotland has implemented intensive programmes to prevent youth violence and reduce drug and alcohol misuse, focussing on vulnerable young people.[62,63] Both England and Scotland implemented the 'challenge 25' policy in 2009 to reduce youth access to alcohol,[64] but Scotland is planning to introduce minimum pricing for alcohol – a move so far resisted in England.(http://www.alcohol-focus-scotland.org.uk/ref)

Implications

Our analyses show that the incidence of MVR injury admissions in children can change substantially over time and in opposite directions in adjacent countries with similar healthcare systems. The declines in Scotland suggest that the increasing rates observed in England are not inevitable. However, which policies, if any, have influenced these changes cannot be determined from this

For peer review only - http://bmjopen.bmj.com/site/about/guidelines.xhtml

study. A priority for future research is to distinguish true change in the occurrence of MVR injury needing admission from changes in coding or admission thresholds. This requires analyses of all cases of MVR injury presenting to primary care, those seen as outpatients by community paediatricians, those attending the emergency department and those admitted to hospital, to understand how children are managed within the healthcare system. Such data linkages are not yet possible due to the lack of well-coded, administrative healthcare databases across health sectors, but are a stated aim of government in England and Scotland.[65,66]

Hospitalisation for maltreatment-related injury or injury due to other forms of victimisation represents considerable suffering to the child and a major cost to the health service. These results strengthen the call by WHO to widen use administrative data to improve understanding of how policy can reduce exposure of children to injury due to violence or neglect.[67] Consideration should also be given to linking survey data of adolescent self-reported exposures to health administrative data to measure service use in children and adolescents exposed to maltreatment or violence.

For peer review only

Licence for publication

The corresponding author has the right to grant on behalf of all authors and does grant on behalf of all authors, an exclusive licence on a worldwide basis to the BMJ Publishing Group Ltd and its licensees, to permit this article (if accepted) to be published in BMJ editions and other BMJPG products and to exploit all subsidiary rights, as set out in our licence (http://resources.bmj.com/bmj/authors/checklists-forms/licence-for-publication)

Competing interests

All authors have completed the Unified Competing Interest form at www.icmje.org/coi_disclosure.pdf (available on request from the corresponding author). AGI, MCB, JW, JM, JMG, JT, CP and RG have no competing interests that may be relevant to the submitted work.

Authors' contributions

RG and AGI conceived the paper and the analytic plan. AGI wrote the first draft and carried out the analyses. RG wrote the final version. All authors commented on the analyses and report. RG is guarantor.

Acknowledgment

AGI was supported by funding from the Department of Health Policy Research Programme through funding to the Policy Research Unit in the Health of Children, Young People and Families. The views expressed are not necessarily those of the Department. We would like to thank members of the Policy Research Unit for the health of children, young people and families: Terence Stephenson, Catherine Law, Amanda Edwards, Ruth Gilbert, Steve Morris, Helen Roberts, Catherine Shaw, Russell Viner and Miranda Wolpert. We are grateful to Andrew Woolley for commenting on the policy timeline for England.

This work is supported by awards establishing the Farr Institute of Health Informatics Research at UCLP Partners from the MRC, in partnership with Arthritis Research UK, the British Heart Foundation, Cancer Research UK, the Economic and Social Research Council, the Engineering and Physical Sciences Research Council, the National Institute of Health Research, the National Institute for Social Care and Health Research (Welsh Assembly Government), the Chief Scientist Office (Scottish Government Health Directorates) and the Wellcome Trust (MR/K006584/1).

Transparency declaration

Ruth Gilbert on behalf of all co-authors affirms that this manuscript is an honest, accurate, and transparent account of the study being reported; that no important aspects of the study have been

For peer review only

For peer review only - http://bmjopen.bmj.com/site/about/guidelines.xhtml

omitted; and that any discrepancies from the study as planned (and, if relevant, registered) have been explained.

Data sharing

Additional data can be accessed in the web-appendix. Source data can be accessed by researchers applying to the Information Services Division Scotland or the Health and Social Care Information Centre for England.

For peer review only

For peer review only - http://bmjopen.bmj.com/site/about/guidelines.xhtml

Reference List

1. World Health Organization. Health Topics. Child maltreatment. 2013. WHO. 2013.
2. HM Government. Working together to safeguard children. A guide to interagency working to safeguard and promote the welfare of children. 2013. London, Department for Education.
3. Finkelhor D, Turner H, Ormrod R, Hamby SL: Violence, abuse, and crime exposure in a national sample of children and youth. Pediatrics 2009, 124: 1411-1423.
4. Radford L, Corral S, Bradley C, Fisher H, Bassett C, Howat N et al.. Child abuse and neglect in the UK today. 2011. London, NSPCC.
5. Finkelhor D, Ormrod RK, Turner HA: Polyvictimization and trauma in a national longitudinal cohort. Dev Psychopathol 2007, 19: 149-166.
6. Finkelhor D, Ormrod RK, Turner HA: The developmental epidemiology of childhood victimization. J Interpers Violence 2009, 24: 711-731.
7. Gonzalez-Izquierdo A, Ward A, Smith P, Walford C, Begent J, Ioannou Y et al.: Notifications for child safeguarding from an acute hospital in response to presentations to healthcare by parents. Child: care health and development, 2013, Under review.
8. Flaherty EG, Sege RD, Griffith J, Price LL, Wasserman R, Slora E et al.: From suspicion of physical child abuse to reporting: primary care clinician decision-making. Pediatrics 2008, 122: 611-619.
9. Gilbert R, Kemp A, Thoburn J, Sidebotham P, Radford L, Glaser D et al.: Recognising and responding to child maltreatment. Lancet 2009, 373: 167-180.
10. World Health Organization Regional Office for Europe. European report on preventing child maltreatment. Edited by Sethi D, Bellis M, Hughes K, Gilbert R, Mitis F, Gauden G. 2013. World Health Organization Regional Office for Europe.
11. Gilbert R, Fluke J, O'Donnell M, Gonzalez-Izquierdo A, Brownell M, Gulliver P et al.: Child maltreatment: variation in trends and policies in six developed countries. Lancet 2012, 379: 758-772.
12. Finkelhor D, Ormrod R, Turner H, Holt M: Pathways to poly-victimization. Child Maltreat 2009, 14: 316-329.
13. De VM, Demyttenaere K, Bruffaerts R: The relationship between adverse childhood experiences and mental health in adulthood. A systematic literature review. Tijdschr Psychiatr 2013, 55: 259-268.
14. Farrington DP, Loeber R: Epidemiology of juvenile violence. Child Adolesc Psychiatr Clin N Am 2000, 9: 733-748.
15. Murray J, Farrington DP: Risk factors for conduct disorder and delinquency: key findings from longitudinal studies. Can J Psychiatry 2010, 55: 633-642.

For peer review only

For peer review only - http://bmjopen.bmj.com/site/about/guidelines.xhtml

16. Norman RE, Byambaa M, De R, Butchart A, Scott J, Vos T: The long-term health consequences of child physical abuse, emotional abuse, and neglect: a systematic review and meta-analysis. PLoS Med 2012, 9: e1001349.

17. Statham J: Effective services to support children in special circumstances. Child Care Health Dev 2004, 30: 589-598.

18. NHS National Services Scotland - Information Services Division. Health and Social Care Data Dictionary - SMR01 General/ Acute Impatient and Day Cases. 2012.

19. NHS.The Information Centre for Health and Social Care. HES Online. What is the data? 2012.

20. Annest JL, Fingerhut LA, Gallagher SS, Grossman DC, Hedegaard H, Johnson RL et al.: Strategies to improve external cause-of-injury coding in state-based hospital discharge and emergency department data systems: recommendations of the CDC Workgroup for Improvement of External Cause-of-Injury Coding. MMWR Recomm Rep 2008, 57: 1-15.

21. Lloyd SS, Rissing JP: Physician and coding errors in patient records. JAMA 1985, 254: 1330-1336.

22. Campbell SE, Campbell MK, Grimshaw JM, Walker AE: A systematic review of discharge coding accuracy. J Public Health Med 2001, 23: 205-211.

23. McKenzie K, Scott D, Waller G, Campbell M: Reliability of Routinely Collected Hospital Data for Child Maltreatment Surveillance. BMC Public Health 2011, 11: 8.

24. McKenzie K, Scott D, Fraser JA, Dunne MP: Assessing the concordance of health and child protection data for maltreated and unintentionally injured children. Inj Prev 2012, 18: 50-57.

25. Schnitzer PG, Slusher PL, Kruse RL, Tarleton MM: Identification of ICD codes suggestive of child maltreatment. Child Abuse Negl 2011, 35: 3-17.

26. Schnitzer PG, Slusher P, Van Tuinen M: Child maltreatment in Missouri: combining data for public health surveillance. Am J Prev Med 2004, 27: 379-384.

27. Gonzalez-Izquierdo A, Ward A, Donnell O, Li L, Roposch A, Stanley F et al.: Cross-country comparison of victimisation-related injury admission in children and adolescents in England and Western Australia. BMC Health Serv Res 2013, 13: 260.

28. Gonzalez-Izquierdo A, Woodman J, Copley L, van der Meulen J, Brandon M, Hodes D et al.: Variation in recording of child maltreatment in administrative records of hospital admissions for injury in England, 1997-2009. Arch Dis Child 2010, 95: 918-925.

29. General Register Office for Scotland. Mid-Year Population Estimates. 2012.

30. Office for National Statistics. Population Estimates for UK, England and Wales, Scotland and Northern Ireland. 2012.

31. Radford L, Corral S, Bradley C, Fisher H, Bassett C, Howat N et al.. Child cruelty in the UK: An NSPCC study into child abuse and neglect over the past 30 years. 2011. London, NSPCC.

For peer review only - http://bmjopen.bmj.com/site/about/guidelines.xhtml

32. Biglan A, Ary D, Wagenaar AC: The Value of Interrupted Time-Series Experiments for Community Intervention Research. Prevention Science 2000, 1: 31-49.

33. Toms JD, Lesperance ML: Piecewise regression: a tool for identifying ecological thresholds. Ecology 2003, 84: 2034-2041.

34. Wagner AK, Soumerai SB, Zhang F, Ross-Degnan D: Segmented regression analysis of interrupted time series studies in medication use research. Journal of Clinical Pharmacy and Therapeutics 2002, 299-309.

35. Hilbe JM: Negative binomial regression, Second Edition edn. Cambridge: Cambridge University Press; 2011.

36. Friedman JH. A variable span scatterplot smoother. Edited by Laboratory for Computational Statistics. 5. 1984. Standford University. Technical Report No. 5.

37. R Development Core Team. R: A language and environment for statistical computing. 2008. Vienna, R Foundation for Statistical Computing.

38. Venables WN, Ripley BD: Modern Applied Statistics with S, Fourth Edition edn. Springer; 2002.

39. Sonderegger DL, Wang HN, Clements WH, Noon BR: Using SiZer to detect thresholds in ecological data. Frontiers in Ecology and Environment 2009, 7: 190-195.

40. Audit Commission. Payment by results assurance framework. 2006. Audit Commission.

41. Jameson S, Reed MR: Payment by results and coding practice in the National Health Service. The importance for orthopaedic surgeons. J Bone Joint Surg Br 2007, 89: 1427-1430.

42. Woodman J, Pitt M, Wentz R, Taylor B, Hodes D, Gilbert R: Performance of screening tests for child physical abuse in Accident and Emergency Departments. Health Technol Assess 2008, 12: iii, xi-xiii 1-95.

43. Woodman J, Freemantle N, Allister J, de LS, Gilbert R, Petersen I: Variation in recorded child maltreatment concerns in UK primary care records: a cohort study using The Health Improvement Network (THIN) database. PLoS One 2012, 7: e49808.

44. Gill PJ, Goldacre MJ, Mant D, Heneghan C, Thomson A, Seagroatt V et al.: Increase in emergency admissions to hospital for children aged under 15 in England, 1999-2010: national database analysis. Arch Dis Child 2013, 98: 328-334.

45. Appleby J: Are accident and emergency attendances increasing? BMJ 2013, 346: f3677.

46. Stafford A, Parton N, Vincent S, Smith C: Child protection systems in the United Kingdom. A comparative analysis. London, UK: Jessica Kingsley Publishers; 2012.

47. Department for Education. Every child matters. Chapter 5 Accountability and integration. 2003. The Stationary Office.

For peer review only - http://bmjopen.bmj.com/site/about/guidelines.xhtml

48. The Scottish Goverment. A guide to Getting it right for every child. Chapter 2 Getting it right in practice. 2012. The Scottish Goverment.

49. Children's Hearings System. Getting it right for every child. Proposals for action. 2005. Scottish Executive.

50. The Scottish Government. Getting it right for children and families. A guide to getting it right for every child. 2012.

51. Kirk CB, Lucas-Herald A, Mok J: Child protection medical assessments: why do we do them? Arch Dis Child 2010, 95: 336-340.

52. Woodman J, Allister J, Rafi I, de Lusignan S, Belsey J, Petersen I et al.: A simple approach to improve recording of concerns about child maltreatment in primary care records: developing a quality improvement intervention. Br J Gen Pract 2012, 62: e478-e486.

53. Woodman J, Gilbert RE, Allister J, Glaser D, Brandon M: Responses to concerns about child maltreatmentt: a qualitative study of GPs in England. BMJ Open 2013, 3, 12. e003894. doi: 10.1136/bmjopen-2013-003894.

54. National Collaborating Centre for Women's and Children's Health. When to suspect child maltreatment . 2009. London, National Institute for Health and Clinical Excellence.

55. Connolly S, Bevan G, Mays N. Funding and performance of healthcare systems in the four UK countries before and after devolution. 2010. London, The Nuffield Trust.

56. Greer S. Four Way Bet: How devolution has led to four different models for the NHS. 2004. London, The Constitution Unit.

57. McGhee J: Children's Hearings in Scotland: Balancing Rights and Welfare. In Social Work and the Law in Scotland. 2nd edition edition. Edited by Davies R, Gordon J. Milton Keynes: The Open University and Basingstoke: Palgrave Macmillan; 2011:85-100.

58. Information Services Division Scotland. Alcohol-related hospital statistics Scotland 2012/13. 2012. NHS Scotland.

59. Office for National Statistics. Crime in England and Wales. 2014.

60. The Scottish Government. High level summary of statistics trend. Crime victimisation. 2011.

61. The Scottish Government. 2010/11 Scottish Crime and Justice Survey: Main findings. 2011.

For peer review only - http://bmjopen.bmj.com/site/about/guidelines.xhtml

62. The Scottish Government. Youth Justice. 2013. The Scottish Goverment. 2013.

63. Gov.UK. Youth crime prevention programmes. 2013. Gov.UK. 2013.

64. Drink Aware. Buying alcohol. 2013. Drink Aware.

65. Health and Social Care Information Centre. Data linkage and extract service. 2014. NHS.

66. Information Services Division Scotland. Electronic data research and innovation service (eDRIS). 2013. NHS Scotland.

67. WHO. World Report on Violence and health. 1-372. 2002.

For peer review only

For peer review only - http://bmjopen.bmj.com/site/about/guidelines.xhtml

Table 1 Hospital admissions for maltreatment or violence-related (MVR) injury in children in England and Scotland between 2005 and 2011, inclusive

Country	Age group	Total unplanned injury	Unplanned injury rates per 100,000 cy *	MVR injury	MVR incidence rates per 100,000cy *	% of total MVR injury**	% of total injury ***
England	<1y	61,987	1361.3	3,955	86.9	7.3	6.4
	1-10y	402,334	962.5	7,876	18.8	14.5	2.0
	11-18y	414,259	1157.4	42,376	118.4	78.2	10.2
	Subtotal	878,580	1069.5	54,207	66.0	100	6.2
Scotland	<1y	5,168	1277.9	290	71.7	3.9	5.6
	1-10y	48,357	1244.3	570	14.7	7.7	1.2
	11-18y	51,339	1471.0	6,507	186.4	88.4	12.7
	Subtotal	104,864	1347.7	7,367	94.7	100	7.0

* Denominators are the mid-year population estimates from Office for National Statistics and General Register Office for Scotland, cy = child years
** Denominator is the total number of maltreatment or violence-related injury admissions in children 0 to 18 years of age by country
*** Denominator is the total number of unplanned injury admissions within age group & country

For peer review only

Table 2 Observed mean incidence rate per 100,000 children in calendar years 2005 and 2011 and absolute difference in rates

		Rate (95%CI) **2005**		Rate (95%CI) **2011**		Absolute difference in rates (95%CI)	
Country	Age group	MVR	Non-MVR	MVR	Non-MVR	MVR	Non-MVR
England	<1y	75.0 (68.1, 81.9)	1146.9 (1120.0, 1173.9)	102.8 (95.2, 110.4)	1334.7 (1307.2, 1362.2)	27.8 (27.1, 28.5)	187.8 (187.2, 188.3)
	1-10y	15.5 (14.5, 16.5)	909.6 (901.9, 917.3)	23.3 (22.1, 24.5)	950.6 (942.9, 958.2)	7.8 (7.6, 8.0)	41.0 (41.0, 40.9)
	11-18y	119.1 (116.2, 122.1)	1076.5 (1067.6, 1085.4)	103.3 (100.6, 106.1)	896.2 (888, 904.5)	-15.8 (-15.6, -16.0)	-180.3 (-179.6, -180.9)
Scotland	<1y	66.1 (44.5, 87.7)	1426.3 (1326.0, 1526.6)	89.6 (65.5, 113.7)	102 4.1 (942.5, 1105.6)	23.5 (21.0, 26.0)	-402.3 (-383.5, -421.0)
	1-10y	19.6 (16.0, 23.3)	1306.2 (1276.2, 1336.3)	9.7 (7.2, 12.3)	1136.6 (1108.8, 1164.3)	-9.9 (-8.8, -11.0)	-169.7 (-167.4, -172.0)
	11-18y	205.8 (193.4, 218.2)	1314.3 (1283.0, 1345.7)	120.6 (110.8, 130.5)	1113.7 (1083.8, 1143.7)	-85.2 (-82.6, -87.7)	-200.6 (-199.2, -202.0)

For peer review only

For peer review only - http://bmjopen.bmj.com/site/about/guidelines.xhtml

Figure 1 Monthly incidence trends from Jan 2005 to Mar 2012 of MVR injury in A) Infants, B) Children aged 1-10 years, and C) Adolescents aged 11-18 years, in England (dark grey**) and Scotland (**light grey**)**. Faint lines represent observed rates and bold lines represent three-monthly moving averages. Dashed lines represent smoothed trends of incidence rates estimated from the segmented regression analysis (except for trends in Scottish 1-10 year-olds where a standard Poisson regression was used) and markers indicate the change point estimated by the segmented regression model.

Figure 2 Absolute rate difference between annual incidence rates in calendar years 2005 and 2011 for MVR injury and non-MVR injury admissions of children and adolescents by age group and country.

Footnote for the figure: % change reflects proportionate change measured as (absolute difference between rate in 2005 and 2011)/(rate in 2005); MVR11-05 = difference in maltreatment or violence related injury admissions between 2005 and 2011 (similarly for non-MVR11-05)

For peer review only - http://bmjopen.bmj.com/site/about/guidelines.xhtml

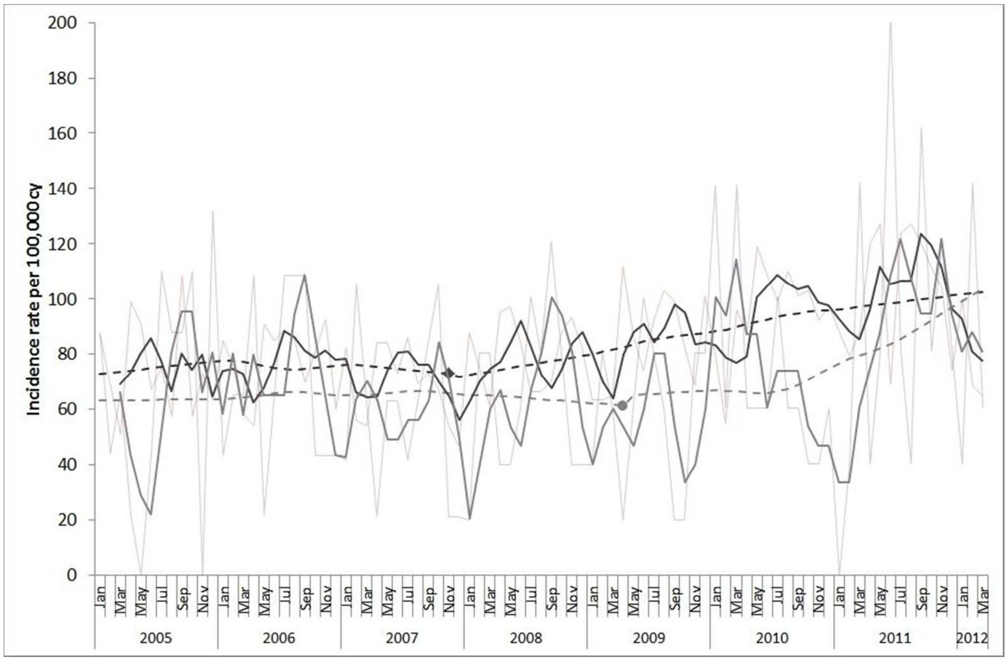

82x53mm (300 x 300 DPI)

eview only

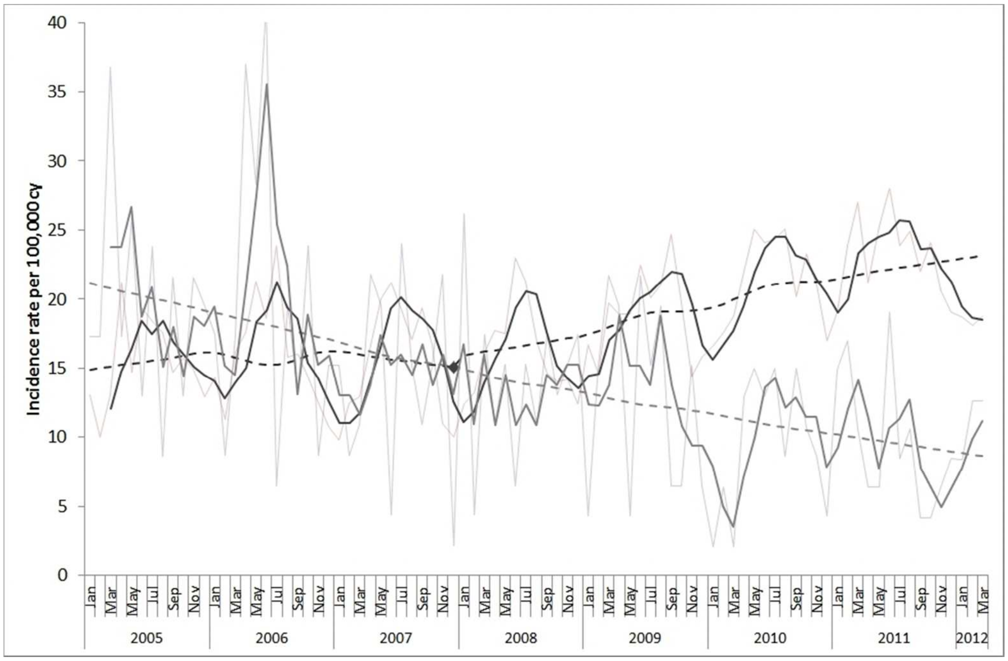

82x53mm (300 x 300 DPI)

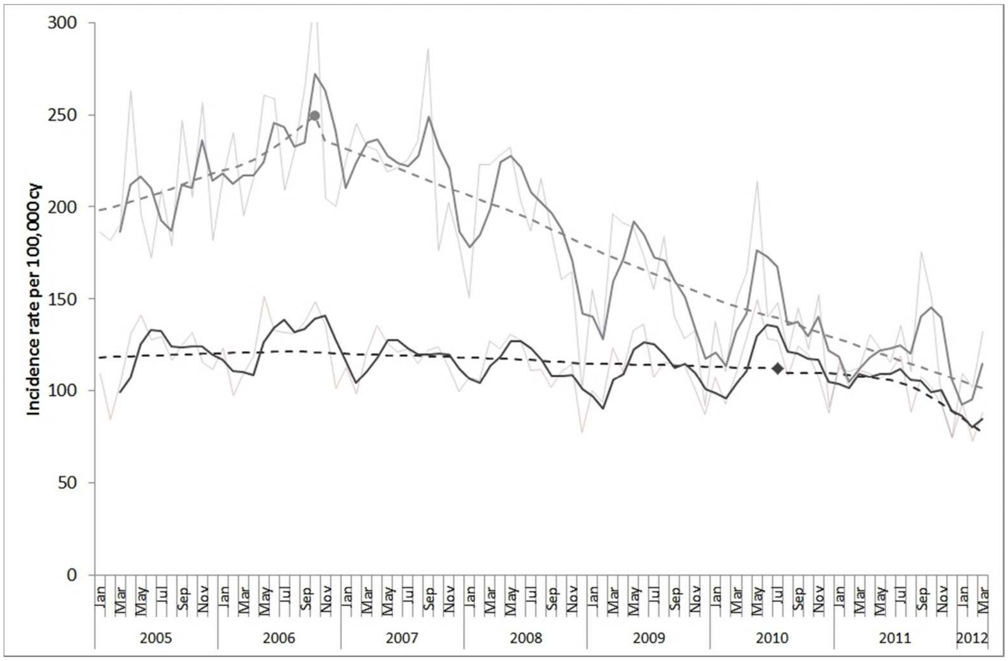

82x53mm (300 x 300 DPI)

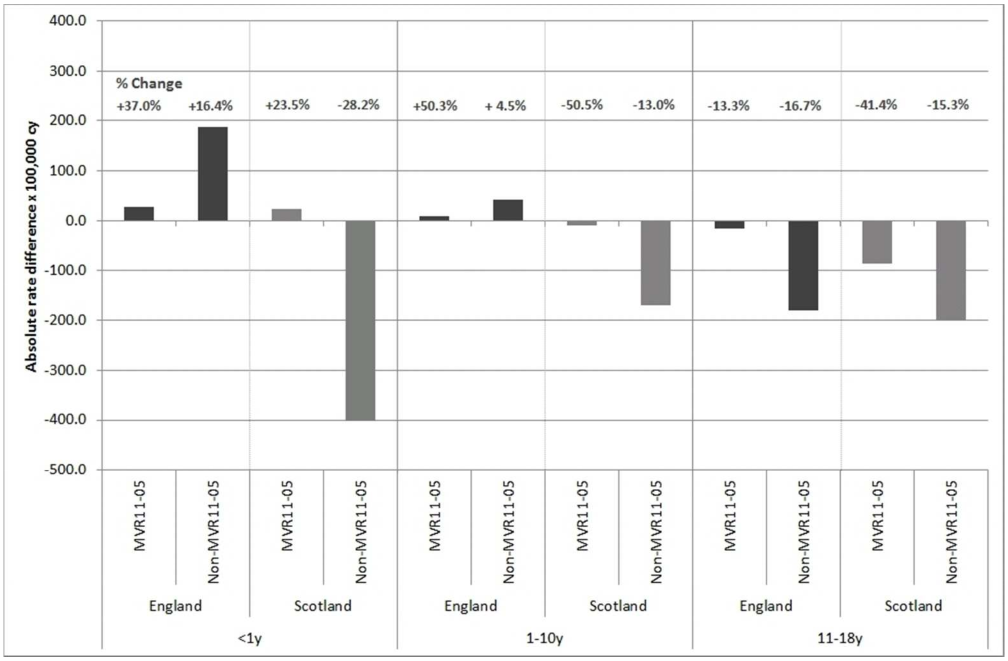

82x53mm (300 x 300 DPI)

For peer review only

WEB-APPENDIX

Web Appendix 1 Segmented negative binomial model with additional terms for non-maltreatment-or-violence-related injury and seasonality terms.

$$y_j \sim Poisson(\mu_j^*)$$

$$\log(\mu_j^*) = \beta_1 + \beta_2 t_j + \beta_3 w_j + \beta_4 z_j + \beta_5 \sin\left(\frac{2\pi.t_j}{12}\right) + \beta_6 \cos\left(\frac{2\pi.t_j}{12}\right) + offset_j + \nu_j$$

$$e^{\nu_j} \sim Gamma\left(1/\alpha, \alpha\right)$$

Where

y = observed count; *j* = *j-th* observation; μ = mean count; t = time,

w = time after estimated change point

z = term for non-maltreatment-or-violence-related injury

sin & cos = terms for seasonality (12-month cycle)

offset = term for population denominator with coefficient = 1

β_1 = Baseline intercept

β_2 = Baseline trend (incidence rate ratio - IRR)

β_3 = Change in trend after estimated change point

β_4 = underlying trend for non-maltreatment related injury

β_5 and β_6 = coefficients for seasonality terms

ν_j is a random effect term with α as the over-dispersion parameter

For peer review only

For peer review only - http://bmjopen.bmj.com/site/about/guidelines.xhtml

Web-Table 1 Definitions and International Classification of Diseases (ICD-10) diagnostic codes used to classify maltreatment or violence-related (MVR) injury

DEFINITIONS

Unplanned admission
An unplanned hospital admission included all longitudinally linked finished consultant episodes (FCE) classified as having an emergency or unplanned method of admission. Admissions included ordinary admissions and day cases. Transfers and injury admissions within 2 days were counted as the same event.

Injury
We classified an admission as injury if it had at least one S or T diagnostic code from the ICD-10 in any FCE

Searching strategy
We looked for injury or MVR diagnostic codes in up to 20 diagnosis fields per FCE in HES or six diagnostic fields in SMR

Exclusions
We excluded babies admitted before 7 days of age to avoid counting injuries most likely to have occurred during birth. Maternity-related admissions were also excluded.

MVR CODES AND DESCRIPTIONS	ICD-10 CODES
**Maltreatment**	
Maltreatment syndromes	T74
Effects of other deprivation (extreme neglect)	T73
Perpetrator of neglect and other maltreatment syndromes	Y06, Y07
**Assault**	
Assault by bodily force and sexual assault	Y04, Y05
Other types of assault	X85 - Y03, Y08 - Y09
**Undetermined cause**	
Events of undetermined intent	Y20 - Y34
Examination and observation following other inflicted injury	Z04.5
Examination and observation for other reasons: request for expert evidence	Z04.8
**Adverse social circumstances**	
Neonatal withdrawal symptoms from maternal use of drugs of addiction	P96.1
Other problems related to physical environment	Z58.8
Problem related to physical environment, unspecified	Z58.9
Homelessness	Z59.0
Inadequate housing	Z59.1
Lack of adequate food	Z59.4
Extreme poverty	Z59.5
Insufficient social insurance and welfare support	Z59.7
Problem related to housing and economic circumstances, unspecified	Z59.9
Problems related to social environment	Z60
Problems related to negative life events in childhood	Z61
Other problems related to upbringing	Z62
Other problems related to primary support group	Z63
Discord with counsellors	Z64.4
Problems related to other legal circumstances	Z65.3
Other specified problems related to psychosocial circumstances	Z65.8
Problem related to unspecified psychosocial circumstances	Z65.9
Problems related to lifestyle	Z72.3 - Z72.9
Problems related to care-provider dependency	Z74
Health supervision and care of foundling	Z76.1
Health supervision and care of other healthy infant and child	Z76.2
Family history of mental and behavioural disorders	Z81
Personal history of other specified risk-factors, not elsewhere classified	Z91.8

For peer review only - http://bmjopen.bmj.com/site/about/guidelines.xhtml

Web-Table 2: Change in gradient of monthly incidence trends in MVR injury in England and Scotland between 2005 and 2011 (negative binomial segmented regression analyses)

Country	Age group	1st gradient - IRR (95%CI)	Annual gradient (%)*	*p*-value for 1st gradient	change point	*p*-value for change in gradient	2nd **or** overall gradient - IRR (95%CI)	Annual gradient (%)*	*p*-value for 2nd or overall gradient
England	1w-<1y	0.998 (0.959, 1.006)	(-2.45)	0.160	November-07	0.015	1.005 (0.903, 1.010)	(6.56)	0.273
	1-10y	1.000 (0.990, 1.006)	(0.21)	0.723	December-07	0.002	1.008 (1.005, 1.011)	(10.44)	0.004
	11-18y	1.000 (0.998, 1.103)	(0.43)	0.073	July-10	<0.001	0.993 (0.947, 1.000)	(-7.54)	<0.001
Scotland	1w-<1y	0.999 (0.983, 1.089)	(-0.80)	0.860	April-09	0.057	1.014 (0.995, 1.074)	(17.98)	0.065
	1-10y					NA	0.991 (0.987, 0.994)	(-10.81)	<0.001
	11-18y	1.009 (0.999, 1.021)	(11.74)	0.003	October-06	<0.001	0.989 (0.986, 0.991)	(-12.87)	0.049

* Effective annual rate = $(IRR^{12} - 1) \times 100$ (calculation before rounding of IRR)

For peer review only - http://bmjopen.bmj.com/site/about/guidelines.xhtml

Web-table 3 Policy initiatives on child protection in England and Scotland 1997 – 2010 potentially having an impact on recognition and recording of child maltreatment, in healthcare

Year	England	Scotland
**1998**	The Data Protection Act 1998 **[L]**	Protecting children – A shared responsibility. Guidance for interagency co-operation **[SG]**
**1999**	Working together to safeguarding children: A guide to interagency working to safeguarding and promote the welfare of children **[SG]**	
**2000**		Protecting children – A shared responsibility. Guidance for health professionals (the pink book) **[HG]**
**2002**	The Adoption and Children Act 2002 **[L]**<br>Safeguarding children in whom illness is fabricated or induced **[SG]**	"It's everyone's job to make sure I'm alright" Report of the child protection audit e review **[S]**
**2003**	What to do if you're worried a child is being abused **[SG]**<br>Every child matters **[S]**<br>Keeping children safe: the government's response to the Victoria Climbié inquiry report and the joint chief inspectors' report Safeguarding Children **[S]**<br>Confidentiality: NHS Code of Practice **[SG]**	Getting our Priorities Right. Good Practice Guidance for working with Children and Families affected by Substance Misuse **[SG]**
**2004**	National service framework for children, young people and maternity services **[IAF]**<br>Every child matters: change for children **[S]**<br>Every child matters: next steps **[S]**<br>Children's and Maternity Services Information Strategy **[HG]**<br>Children Act 2004 **[L]**	Protecting children and young people: framework for standards **[IAF]**
**2005**	Common core of skills and knowledge for the children's workforce **[SG]**	Scottish Executive. Getting it right for every child **[S]**
**2006**	Working together to safeguarde children. **[SG]**<br>What to do if you're worried a child is being abused **[SG]**	Joint inspection of children's services and inspection of social work services (Scotland) Act 2006 **[L]**
**2007**	The 2007 Pre-budget Report and Comprehensive Spending Review **[IAF]**<br>Safeguarding children who may have been trafficked **[SG]**<br>Safeguarding children from abuse linked to a belief in spirit possession **[SG]**<br>Statutory guidance on making arrangements to safeguard and promote the welfare of children under section 11 of the Children Act 2004 **[SG]**	Protecting children and young people: Interim guidance for child protection committees for conducting a significant case review **[SG]**<br>Protection of vulnerable groups (Scotland) Act 2007 **[L]**
**2008**	The right to choose: Multi-agency statutory guidance for dealing with forced marriage **[SG]**<br>Safeguarding children in whom illness is fabricated or induced (revised) **[SG]**	A guide to implementing Getting it right for every child **[IAF]**<br>National domestic abuse delivery plan for children and young people **[S]**<br>The early years framework **[IAF]**

For peer review only - http://bmjopen.bmj.com/site/about/guidelines.xhtml

For peer review only

**2009**	NICE guidelines on when to suspect child maltreatment **[SG]**<br>Statutory guidance on promoting the health and well-being of looked after children **[SG]**<br>Safeguarding children and young people from sexual exploitation **[SG]**<br>Confidentiality: Reporting knife and gunshot wounds (supplementary guidance) **[HG]**<br>Safeguarding children: a review of arrangements in the NHS for safeguarding children **[IAF]**<br>The protection of children in England: action plan: The Government's response to Lord Laming **[S]**<br>The CQC (Registration) Regulations **[IAF]**	Sexual offences (Scotland) Act 2009 **[L]**<br>Safeguarding children in Scotland who may have been trafficked **[SG]**
**2010**	Working together to safeguarding children. A guide to interagency working to safeguarding and promote the welfare of children **[SG]**<br>NICE guidance on pregnant women with social factors **[SG]**	The national guidance for child protection in Scotland **[SG]**

Policy initiative selection criteria: We included public initiatives likely to have an impact on recognition and recording of child maltreatment, in health care. They had to be legislative acts **[L]**, statutory or inter-agency guidance **[SG]**, government strategies **[S]**, implementation or accountability frameworks **[IAF]**, or health guidance **[HG]** that applied at a national level. We excluded research or evidence synthesis and guidance published by professional bodies.

For peer review only

For peer review only - http://bmjopen.bmj.com/site/about/guidelines.xhtml

Web-figure 1: Monthly incidence trends from Jan 2005 to Mar 2012 of non-MVR injury in A) Infants, B) Children aged 1-10 years, and C) Adolescents aged 11-18 years, in England (dark grey**) and Scotland (**light grey**).** Faint lines represent observed rates and bold lines represent three-monthly moving averages

A)

B)

C)

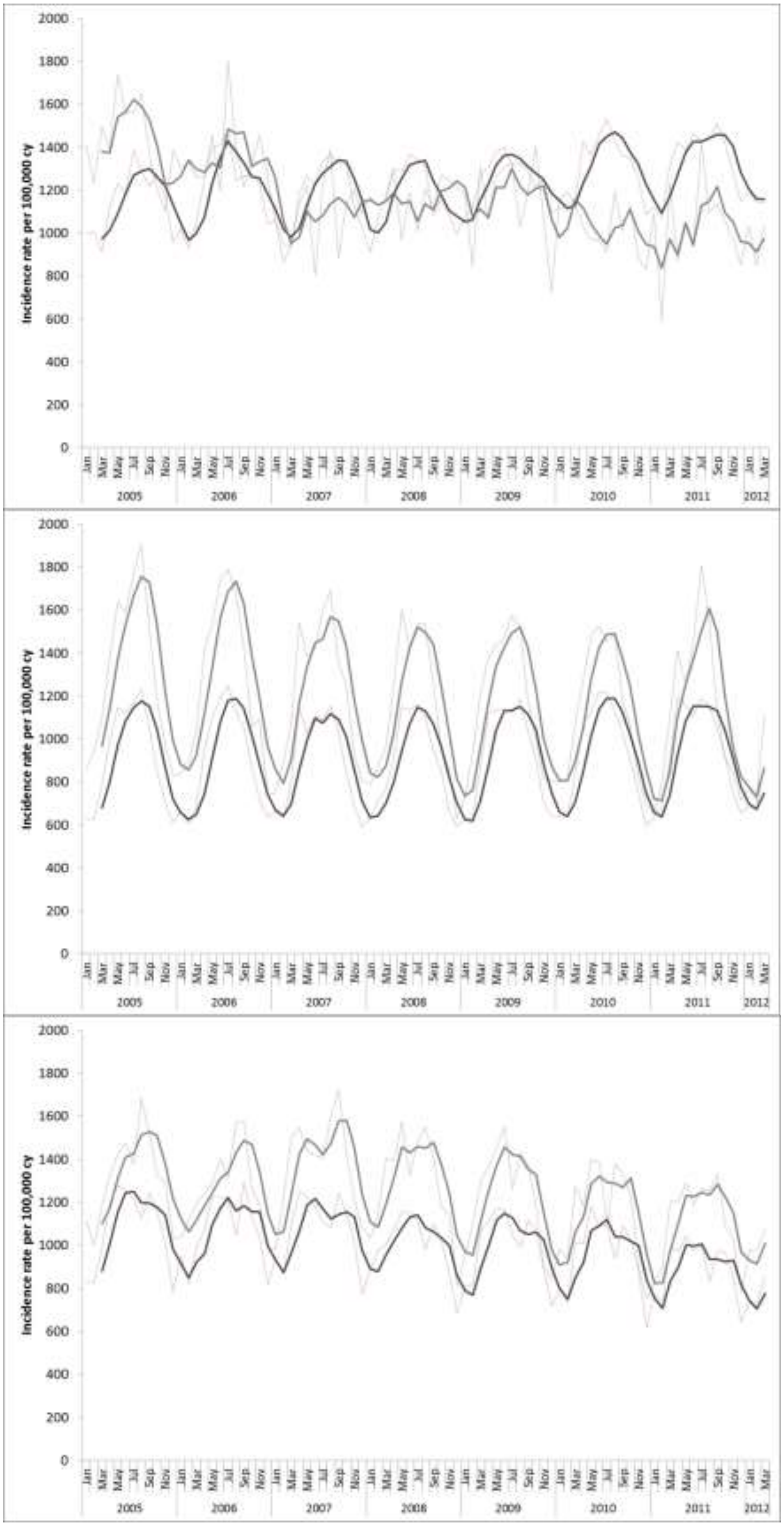

Supplement: Author's manuscript [file bmjopen-2013-004474.draft_revisions.pdf]
